# Characterization update of HIV-1 M subtypes diversity and proposal for subtypes A and D sub-subtypes reclassification

**DOI:** 10.1186/s12977-018-0461-y

**Published:** 2018-12-22

**Authors:** Nathalie Désiré, Lorenzo Cerutti, Quentin Le Hingrat, Marine Perrier, Stefan Emler, Vincent Calvez, Diane Descamps, Anne-Geneviève Marcelin, Stéphane Hué, Benoit Visseaux

**Affiliations:** 1 INSERM, Institut Pierre Louis d’Epidémiologie et de Santé Publique (iPLESP), Sorbonne Université, 75013 Paris, France; 2 AP-HP, Hôpital Pitié-Salpêtrière, Virologie, 75013 Paris, France; 3 SmartGene Services, EPFL Innovation Park, 1015 Lausanne, Switzerland; 4 IAME, UMR 1137, Université Paris Diderot, INSERM, Paris, France; 5 AP-HP, Hôpital Bichat, Virologie, 75018 Paris, France; 6 Department of Epidemiology & Population Health, London School of Hygiene and Tropical Medicine, London, WC1E 7HT UK

**Keywords:** HIV, Nomenclature, Viral diversity, Phylogenetic, Evolution

## Abstract

**Background:**

The large and constantly evolving HIV-1 pandemic has led to an increasingly complex diversity. Because of some taxonomic difficulties among the most diverse HIV-1 subtypes, and taking advantage of the large amount of sequence data generated in the recent years, we investigated novel lineage patterns among the main HIV-1 subtypes.

**Results:**

All HIV full-length genomes available in public databases were analysed (n = 2017). Maximum likelihood phylogenies and pairwise genetic distance were obtained. Clustering patterns and mean distributions of genetic distances were compared within and across the current groups, subtypes and sub-subtypes of HIV-1 to detect and analyse any divergent lineages within previously defined HIV lineages. The level of genetic similarity observed between most HIV clades was deeply consistent with the current classification. However, both subtypes A and D showed evidence of further intra-subtype diversification not fully described by the nomenclature system at the time and could be divided into several distinct sub-subtypes.

**Conclusions:**

With this work, we propose an updated nomenclature of sub-types A and D better reflecting their current genetic diversity and evolutionary patterns. Allowing a more accurate nomenclature and classification system is a necessary step for easier subtyping of HIV strains and a better detection or follow-up of viral epidemiology shifts.

**Electronic supplementary material:**

The online version of this article (10.1186/s12977-018-0461-y) contains supplementary material, which is available to authorized users.

## Background

HIV-1 presents an extraordinary degree of genetic diversity and has been classified, based on phylogenetic clustering, into groups, subtypes and sub-subtypes. While groups correspond to distinct lineages independently introduced into the human population from non-human apes, subtypes and sub-subtypes results from post-introduction founder events and further diversification. These distinctions have led to the formal recognition of 4 groups (M, N, O and P), 9 group M subtypes (A, B, C, D, F, G, H, J and K) and several sub-subtypes (A1, A2 for subtype A and F1, F2 for subtype F) [1].

HIV classification is used routinely by all medical laboratories performing genotyping resistance testing to characterize patient’s strain, to detect reinfection and to analyse viral epidemiology shifts [2, 3]. HIV subtypes can also present different antiretroviral drug or vaccine response [4–6] [7, 8], disease progression [9–11] or transmission rates [12–14]. All of these observations rely on an up-to-date and representative classification system, reflecting as best as possible the true and constantly evolving diversity of HIV-1 strains. Thus, since HIV-1 is rapidly and continuously evolving, it is important to keep track of the diversification of the virus on a regular basis.

Due to some routinely experienced classification difficulties, particularly with subtype A for which only sub-subtypes A1 and A2 were formally retained before this work, we reanalysed the global HIV-1 group M diversity among all subtypes, at exclusion of recombinant forms, using all available near full genome sequences and combining a systematic phylogenetic distance analysis with maximum likelihood phylogenetic analyses. We identified significant divergence patterns not fully included in the classification at the time among two subtypes, A and D. Thus, we propose a new sub-subtypes classification for these two subtypes to better describe their diversity and provide a more accurate description of their currently observed genetic diversity.

## Methods

### Sequence data

All HIV-1 groups M, N, O and P near full-genome sequences with available subtype information were downloaded from the Los Alamos National Laboratory (LANL) database on June 2016. All LANL partial *gag*, *pol* and *env* gene sequences of subtypes A and D were also collected in specific datasets. CRF (circulating recombinant form) sequences as well as clonal or iterative sequences were excluded from the analysis. As the A3 and A4 sub-subtype were not formally retained into the LANL classification at the time of this analysis, all subtype A sequences were manually re-annotated according to the corresponding publications [15, 16]. The country of sampling was also retrieved for each included sequence.

### Alignments

After removing LTR regions, sequence datasets were aligned using Mafft version 7 with default settings [17] and manually edited using Aliview [18]. Potential recombinants were identified using the package RDP4 [19] and excluded. All genome regions that could not be unequivocally aligned (e.g. highly variable *env* regions) were removed from the alignments. All sequences retained in the final are depicted in Additional file 1: Table S1.

### Pairwise genetic distance calculations

Pairwise genetic distances were calculated using HyPhy 2.2.4 with the GTR model of nucleotide substitutions and a gamma distribution with 4 parameters [20]. This model was chosen using jModelTest 2 [21]. The distributions of near full genome pairwise genetic distance within and between known groups, subtypes and sub-subtypes were plotted using R 3.3.2 and ggplot2 package [22] to describe genetic distance ranges and net genetic divergence observed for intergroup, intersuptypes, intersub-subtypes and intrasub-subtypes comparisons.

### Phylogenetic analyses

In case of large genetic distribution for a particular subtype or sub-subtype, compatible with the existence of various sub-subtypes not retained in the current HIV nomenclature, neighbour joining and maximum likelihood phylogenies were reconstructed using PhyML 3.0 [23] using the GTR model of nucleotide substitutions with a gamma distribution with 4 parameters (GTR-G). Maximum likelihood phylogenies were reconstructed using PhyML 3.0 [23] with GTR-G. An initial NJ tree calculated by default by PhyML was used with both NNI (Next Neighbour Inversion) and SPR (Subtree Pruning and Regrafting) for the tree-space exploration. Branch support was estimated by bootstrap analysis with 1000 replicates. Observed clades strongly supported (i.e. above 90%) and presenting genetic distance in the range of sub-subtype area according to our analysis were used to propose a better classification of these subtypes.

According to current guidelines, a new lineage must include three non-directly linked viral isolates and at least two must be a full genome sequence to be retained as a new subtype or sub-subtype [1]. Thus, when a single near full genome sequence was suggestive of a previously non-described sub-subtype, phylogenies of all available *gag*, *pol* and *env* sequences were used to identify if sequences from other isolates of the same clade were available.

## Results

A total of 2017 near full genome sequences were included in the study, representing 204, 1185, 488, 66, 29, 39, 4, 3 and 2 sequences for A, B, C, D, F, G, H, J and K subtypes, respectively.

**Fig. 1:**
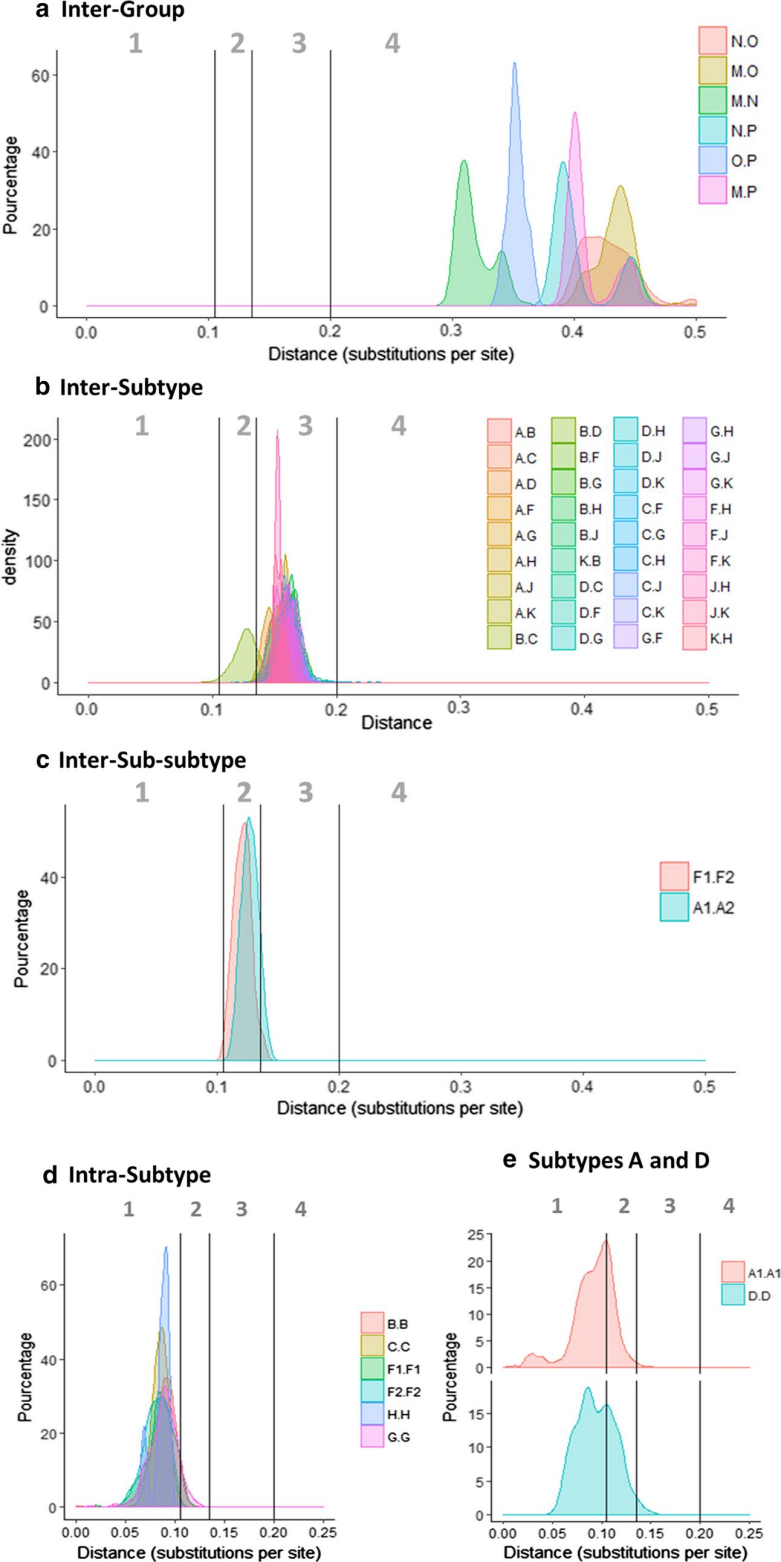
Genetic distance comparisons between HIV-1 groups, subtypes and sub-subtypes using near full genome sequences. X-axis scale lines indicate genetic distance thresholds allowing, in our alignment and model conditions, for group, subtype and sub-subtype identification. Genetic distance ranges compatible with intra-sub-subtype, inter-sub-subtype, inter-subtype and inter-group comparisons are indicated by the numbers 1, 2, 3 and 4, respectively

Near full genome pairwise genetic distance distributions showed patterns consistent with the classification at the time, delimiting ranges of distances within which inter-group, inter-subtypes, inter-sub-subtypes and intra-sub-subtypes genetic diversities almost consistently fall (Fig. 1a–d). Indeed, using these ranges observed in our dataset, two clades, A1 and D, were identified as presenting intra-clade genetic distances inconsistent with the range expected from their respective classification. This highlighted their large diversity and the existence of distinct sub-subtypes within both of them (Fig. 1e). To note, and as previously described, genetic distances observed between subtypes B and D were smaller than those observed between all others inter-subtype comparisons but felt within the range of inter-sub-subtype comparisons (Fig. 1b), confirming that they should represent two sub-subtypes of a single subtype.

**Fig. 2:**
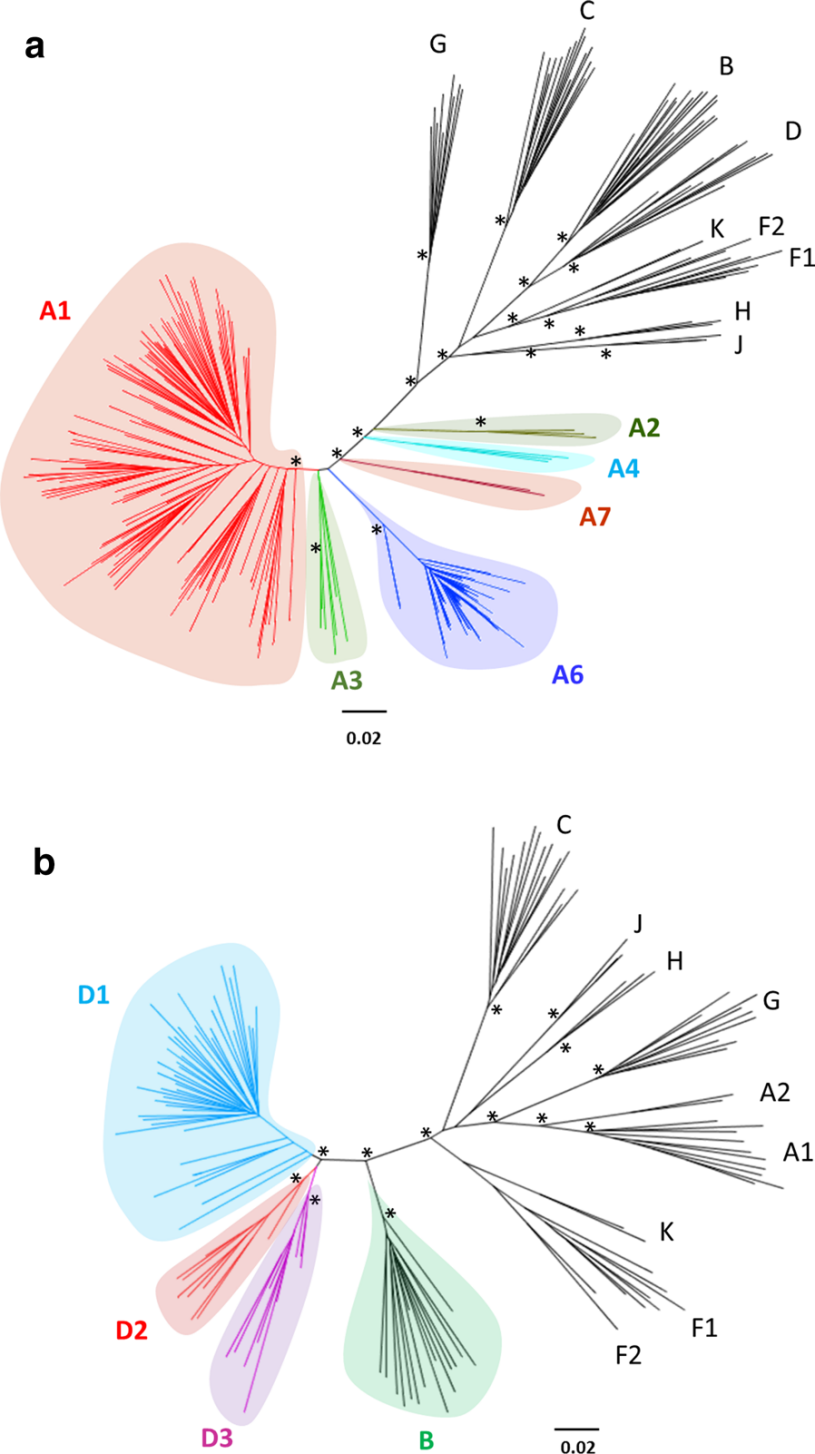
Subtypes A (**a**) and D (**b**) near full genome sequences maximum likelihood phylogenetic trees. Names of all sub-subtypes are indicated according to the new classification proposal. For all identified sub-subtypes and the other major clades, i.e. subtypes, nodes presenting a branch support > 95% (bootstrap analysis using 1000 replicates) are indicated by an asterisk

To assess possible classification improvements within subtype A, a phylogeny of all subtype A full genome sequences was constructed and is shown in Fig. 2a. Several sub-subtypes candidates were observed: the well-established A1 and A2 sub-subtypes, the described but previously not formally retained A3 and A4 sub-subtypes, the former FSU-cluster (herein called A6) and a well separated cluster of 2 sequences (herein called A7). All these clades were supported by a strong branch support at 100%. Four sequences also clustered with the A3 sub-subtypes but were poorly supported (branch support of 3%) and could not be unambiguously included into A3 nor any of the observed clades in the near full genome nor *gag*, *pol* and *env* specific phylogenies. The same tree topology was observed with both neighbour-joining phylogenetic and maximum likelihood reconstruction. To assess if A3 and A4 not formally retained sub-subtypes, as well as FSU-cluster (herein renamed A6) and the newly described A7 clade, all felt in the range of formally retained sub-subtypes, near full genome pairwise genetic distances were calculated. Their distribution supported all these A sub-subtype as candidates with the exception of the A3 sub-subtype presenting a genetic distance distribution with A1 slightly lower than other sub-subtypes (Additional file 1: Fig. S1, Tables S2 and S3). The same patterns of phylogenetic tree topology and genetic distance distributions were also observed when analysing the *gag*, *pol* and *env* genes separately.

Concerning the A7 clade, as only two near full genome sequences are currently available and as at least three sequences, including two full genome sequences, are required to describe a new sub-subtype [1], all available partial *pol* (1329 bp), *gag* (1466 bp) and *env* (2566 bp) genes from subtype A were retrieved from the LANL database (n = 244, 387 and 399 sequences, respectively) and corresponding phylogenies were constructed. Four additional sequences were identified as belonging to the A7 clade, one from the *pol* tree (DQ273915; Additional file 1: Fig. S2) and three from the *gag* tree (Accession number JF683739, EU673432, EU673431; Additional file 1: Fig. S3), allowing to retain A7 as a new sub-subtype.

All these sub-subtypes presented slightly different geographical dispersal as A1 strains were mostly sampled in Eastern Africa from Kenya, Uganda, Tanzania, Rwanda and Cyprus (113/132); A2 in Cyprus, Eastern and Central Africa(3/3); A3 in Senegal (3/3), A4 in Democratic Republic of Congo (3/3); A6 in former Soviet Union countries (Russia, Ukraine, Uzbekistan, etc.) (44/52); A7 in Nigeria for strains identified with the complete genome analysis (2/2), in Cyprus for strains identified with gag gene (3/3) and in Nigeria for strains identified with pol gene (1/1).

To assess if distinct sub-subtypes can be identified within the subtype D, the phylogenetic tree obtained for subtype D near full-length genome sequences was constructed and is shown in Fig. 2b. Within subtype D, characterised by a high genetic diversity in the same range than for subtype A, three main clades were observed, herein called D1, D2 and D3 (branch support for 100% for all of them). The proposed D1 clade is composed of a large clade of sequences with a few outsider groups of sequences located nearer of the most recent common ancestor of the D subtype. The same tree topology was observed with both neighbour-joining phylogenetic and maximum likelihood reconstruction. Most of these outsider sequences were sampled in Uganda as for the major clade of D1 and, thus, were also retained into the proposed D1 clade for genetic distance analysis. Genetic distances observed between D1, D2 and D3 sub-subtype candidates showed large distributions, largely compatible with inter-sub-subtype range but also overlapping the intra-sub-subtype range (Additional file 1: Fig. S4 and Tables S2, S3). This large distribution is explained by the few outsider sequences located nearer to the D common ancestor. No other classification choices provided better genetic distance distribution and outsider sequences always presented very close genetic distance to the main groups, preventing to process them as separate sub-subtypes. Despite these outsider sequences, and because of the large genetic distance observed between the three D sub-subtype candidates, we propose to include D1, D2 and D3 into the HIV nomenclature. The same patterns of phylogenetic tree topology and genetic distance distributions were also observed when analysing the *gag*, *pol* and *env* genes separately.

These three sub-subtype candidates presented distinct geographic distribution as D1 strains were sampled in Eastern Africa, mostly in Uganda (38/46); D2 was sampled in Democratic Republic of Congo, South Africa and Brazil (10/10); D3 was mostly sampled in Cameroun, Chad and Republic Democratic of Congo (7/10).

All these observations have also been confirmed by separate *gag*, *pol* and *env* genes for both subtypes A and D (Additional file 1: Figs. S5, S6, S7). However, genetic distance observed for *pol* with the subtype D, if within the range of inter-sub-subtypes comparisons, were not as well separated than with the other genes or full genome analyses (Additional file 1: Fig. S8).

## Discussion

The large HIV genetic diversity is a challenging reflection of HIV complex and changing epidemiology leading to an evolving nomenclature and sometime unresolved issues. Given the amount of sequence data regularly and increasingly generated in the recent years, we re-investigated HIV-1 genetic diversity of all subtypes initially to resolve some issues within subtype A. We propose to slightly modify the subtype A classification by dividing it into 6 sub-subtypes, called A1, A2, A3, A4, A6 and A7. We also propose to divide the highly diverse subtype D into 3 sub-subtypes named D1 to D3.

Thus, at the time of this work, subtype A was formally divided into only two sub-subtypes A1 and A2 and two other sub-subtypes, A3 and A4, were also proposed but not formally retained in the Los Alamos National Laboratory nomenclature. A partially described A5 sub-subtype has also been previously described based on analysis of recombinant viruses but no prototype of this strain has been identified to date [24]. In our analysis, A3 and A4 confirmed to have a degree of diversity compatible with sub-subtypes definition. Interestingly, the Former Soviet Union (FSU) cluster, resulting from the introduction of HIV-1 subtype A in intravenous drug users population of the former Soviet Union countries in the 80s [25], also depicted a high degree of diversity compatible with sub-subtype and, thus, is proposed in this work as a separate sub-subtype called A6. Before the presentation of this part of the current study at the International AIDS Society conference 2017, A2, A3 and the FSU cluster were only identified as sub-subtype A1 by available subtyping tools. Since then, A3, A4 and A6 sub-subtypes have been formally implemented into the Los Alamos National Laboratory HIV database and allow a quick and more accurate description of the subtype A diversity. In this work we also added the description of the A7 sub-subtype that was not previously described. We also enlarged the analysis to all other HIV-1 subtypes and identified evidence of further diversification consistent with distinct sub-subtypes among the subtype D. Thus, 3 sub-subtypes are proposed for this latter subtype, called D1 to D3, allowing a better description of D diversity despite the existence of a few outsider sequences. Their classification may have to continue to evolve in the future depending of the potential acquisition of new sequences.

Due to the constant evolution of HIV and regular addition of new sequences in public databases, uncertainties about HIV-1 classification have regularly emerged. For example, the previous classification proposal did no resolve whether subtypes F and K represented two different subtypes or sub-subtypes [1]. Our results support the notion that they represent two different subtypes, since they exhibit genetic distances within in the range of other inter-subtype comparisons. A few previous studies also reported large genetic diversity among existing subtypes, as for subtype C [26, 27]. However, in our current work we did not find enough genetic variations within any other subtypes than subtypes A and D to define specific clades according to the current classification rules.

**Fig. 3:**
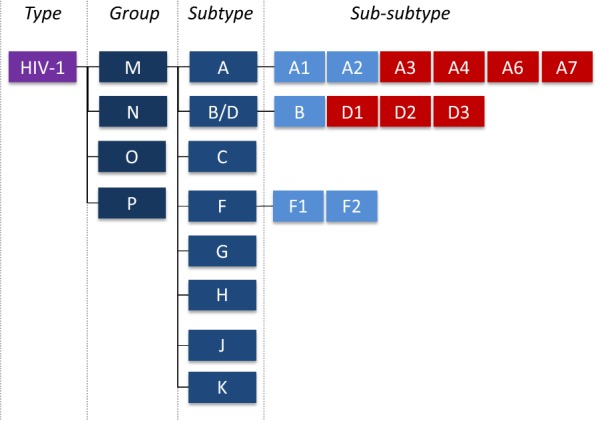
HIV-1 updated classification proposal. HIV-1 remains classified into groups, subtypes and sub-subtypes, with changes from the previous classification colored in red

The classification changes proposed in the current work (Fig. 3) help to provide an up-to-date description of HIV-1 group M heterogeneity and, if some challenges remains, will help to more accurate descriptions of HIV-1 diversification and to keep a better track of HIV-1 upcoming evolutions.

## Additional file

**Additional file 1: Fig. S1.** Full genome genetic distance comparisons between HIV-1 subtype A sub-subtypes according to our classification proposal. X-axis scale lines indicate genetic distance thresholds allowing, in our alignment and model conditions, for group, subtype and sub-subtype identification. **Fig. S2.** Phylogenetic tree of sub-type A obtained with *pol* gene. Sequences from A1, A2, A3, A4 and A6 clades have been collapsed for readability. One *pol* sequence was identified in the A7 clade in addition to the two full genome sequences and is highlighted by an arrow. The tree has been obtained with PhyML 3.0, using GTR-G nucleotide substitution model and branch support obtained by bootstrap method is given for each node. Several sequences, depicted in black, clustered outside the defined clades but cannot be retained in the classification proposal because of poor branch support values or absence of available full genome sequences. **Fig. S3.** Phylogenetic tree of sub-type A obtained with *gag* gene. Sequences from A1, A2, A3, A4 and A6 clades have been collapsed for readability. One pol sequence was identified in the A7 clade in addition to the two full genome sequences and is highlighted by an arrow. The tree has been obtained with PhyML 3.0, using GTR-G nucleotide substitution model and branch support obtained by bootstrap method is given for each node. Several sequences, depicted in black, clustered outside the previously defined clades but cannot be retained in the classification proposal because of poor branch support values or absence of available full genome sequences. **Fig. S4.** Genetic distance comparisons between HIV-1 subtype D sub-subtypes according to our new classification proposal. X-axis scale lines indicate genetic distance thresholds allowing, in our alignment and model conditions, for group, subtype and sub-subtype identification. **Fig. S5.** Genetic distance comparisons between HIV-1 groups, subtypes and sub-subtypes using *pol* sequences. X-axis scale lines indicate genetic distance thresholds allowing, in our alignment and model conditions, for group, subtype and sub-subtype identification. Genetic distance ranges compatible with intra-sub-subtype, inter-sub-subtype, inter-subtype and inter-group comparisons are indicated by the numbers 1, 2, 3 and 4, respectively. **Fig. S6.** Subtypes A (A) and D (B) *pol* sequences maximum likelihood phylogenetic trees. Names of all sub-subtypes are indicated according to the new classification proposal. **Fig. S7.** *Pol* gene genetic distance comparisons between HIV-1 subtype A sub-subtypes according to our classification proposal. X-axis scale lines indicate genetic distance thresholds allowing, in our alignment and model conditions, for group, subtype and sub-subtype identification. **Fig. S8.** *Pol* gene genetic distance comparisons between HIV-1 subtype D sub-subtypes according to our classification proposal. X-axis scale lines indicate genetic distance thresholds allowing, in our alignment and model conditions, for group, subtype and sub-subtype identification. **Table S1.** Full genome sequence used for our analysis and the corresponding clade in our classification proposal. **Table S2.** Full genome genetic distance distributions observed within each corresponding clade. **Table S3.** Net genetic divergence between each identified sub-subtypes. The “net genetic divergence” between each identified sub-subtypes within corresponding subtypes, which also takes into account the within-sub-subtype diversity, was calculated as follow: if d_x,y_ is the average genetic divergence between two sub-subtypes, x and y, and d_x_ and d_y_ are the genetic diversities within populations x and y, respectively, net divergence, D_x,y_, is given by the expression D_x,y_ = d_x,y_ − (d_x_ + d_y_)/2.
