# Supplementary material for: Characterization update of HIV-1 M subtypes diversity and proposal for subtypes A and D sub-subtypes reclassification
Source: Retrovirology. 2018 Dec 22;15:80. doi: 10.1186/s12977-018-0461-y (PMC6303845; doi:10.1186/s12977-018-0461-y)
Supplement: Supplementary file 1 — Additional file 1: Fig. S1. Full genome genetic distance comparisons between HIV-1 subtype A sub-subtypes according to our classification proposal. X-axis scale lines indicate genetic distance thresholds allowing, in our alignment and model conditions, for group, subtype and sub-subtype identification. Fig. S2. Phylogenetic tree of sub-type A obtained with pol gene. Sequences from A1, A2, A3, A4 and A6 clades have been collapsed for readability. One pol sequence was identified in the A7 clade in addition to the two full genome sequences and is highlighted by an arrow. The tree has been obtained with PhyML 3.0, using GTR-G nucleotide substitution model and branch support obtained by bootstrap method is given for each node. Several sequences, depicted in black, clustered outside the defined clades but cannot be retained in the classification proposal because of poor branch support values or absence of available full genome sequences. Fig. S3. Phylogenetic tree of sub-type A obtained with gag gene. Sequences from A1, A2, A3, A4 and A6 clades have been collapsed for readability. One pol sequence was identified in the A7 clade in addition to the two full genome sequences and is highlighted by an arrow. The tree has been obtained with PhyML 3.0, using GTR-G nucleotide substitution model and branch support obtained by bootstrap method is given for each node. Several sequences, depicted in black, clustered outside the previously defined clades but cannot be retained in the classification proposal because of poor branch support values or absence of available full genome sequences. Fig. S4. Genetic distance comparisons between HIV-1 subtype D sub-subtypes according to our new classification proposal. X-axis scale lines indicate genetic distance thresholds allowing, in our alignment and model conditions, for group, subtype and sub-subtype identification. Fig. S5. Genetic distance comparisons between HIV-1 groups, subtypes and sub-subtypes using pol sequences. X-axis scale line [file 12977_2018_461_MOESM1_ESM.docx]

Fig. S1. Full genome genetic distance comparisons between HIV-1 subtype A sub-subtypes according to our classification proposal. X-axis scale lines indicate genetic distance thresholds allowing, in our alignment and model conditions, for group, subtype and sub-subtype identification.


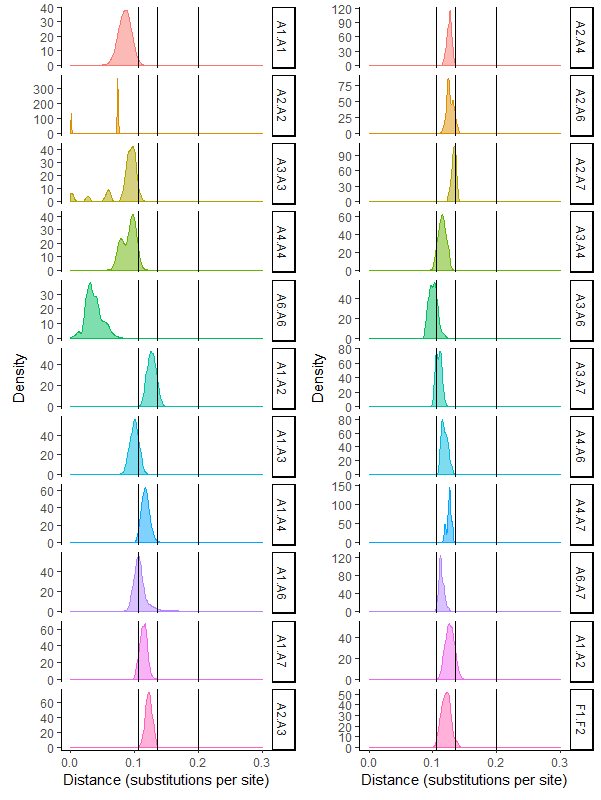


**Fig. S2. Phylogenetic tree of sub-type A obtained with *pol* gene.** Sequences from A1, A2, A3, A4 and A6 clades have been collapsed for readability. One *pol* sequence was identified in the A7 clade in addition to the two full genome sequences and is highlighted by an arrow. The tree has been obtained with PhyML 3.0, using GTR-G nucleotide substitution model and branch support obtained by bootstrap method is given for each node. Several sequences, depicted in black, clustered outside the defined clades but cannot be retained in the classification proposal because of poor branch support values or absence of available full genome sequences.


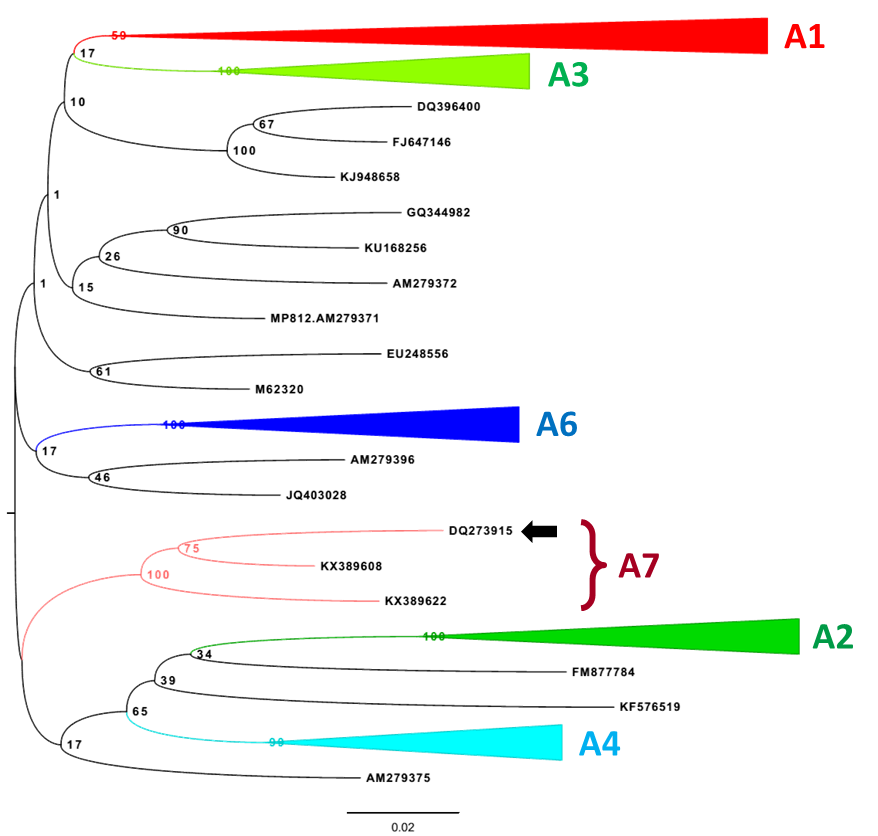


Fig. S3. Phylogenetic tree of sub-type A obtained with *gag* gene. Sequences from A1, A2, A3, A4 and A6 clades have been collapsed for readability. One pol sequence was identified in the A7 clade in addition to the two full genome sequences and is highlighted by an arrow. The tree has been obtained with PhyML 3.0, using GTR-G nucleotide substitution model and branch support obtained by bootstrap method is given for each node. Several sequences, depicted in black, clustered outside the previously defined clades but cannot be retained in the classification proposal because of poor branch support values or absence of available full genome sequences.


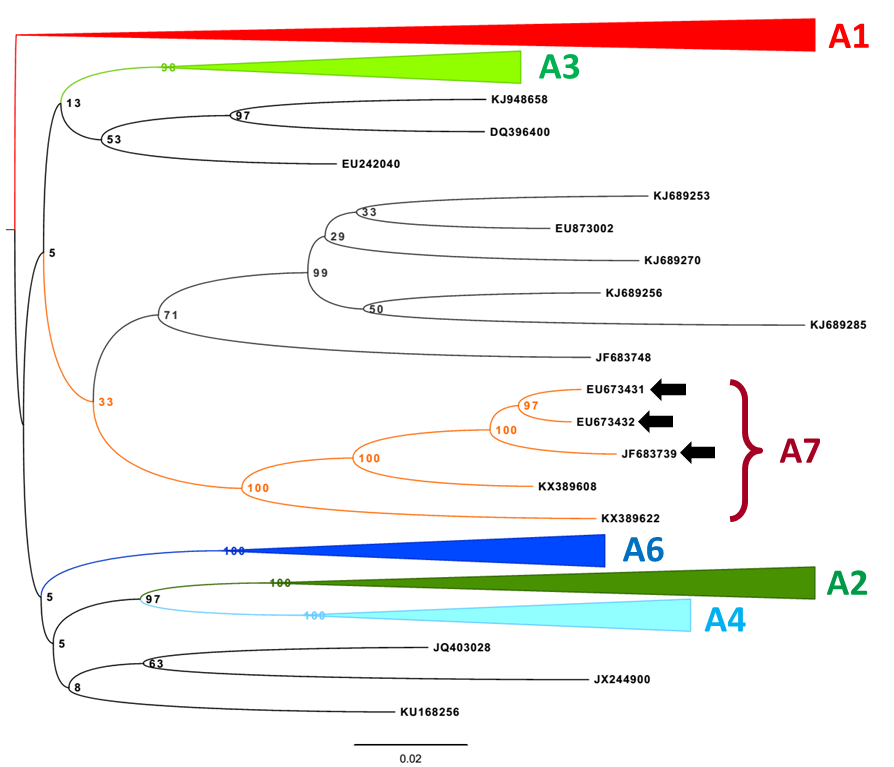


Fig. S4. Genetic distance comparisons between HIV-1 subtype D sub-subtypes according to our new classification proposal. X-axis scale lines indicate genetic distance thresholds allowing, in our alignment and model conditions, for group, subtype and sub-subtype identification.


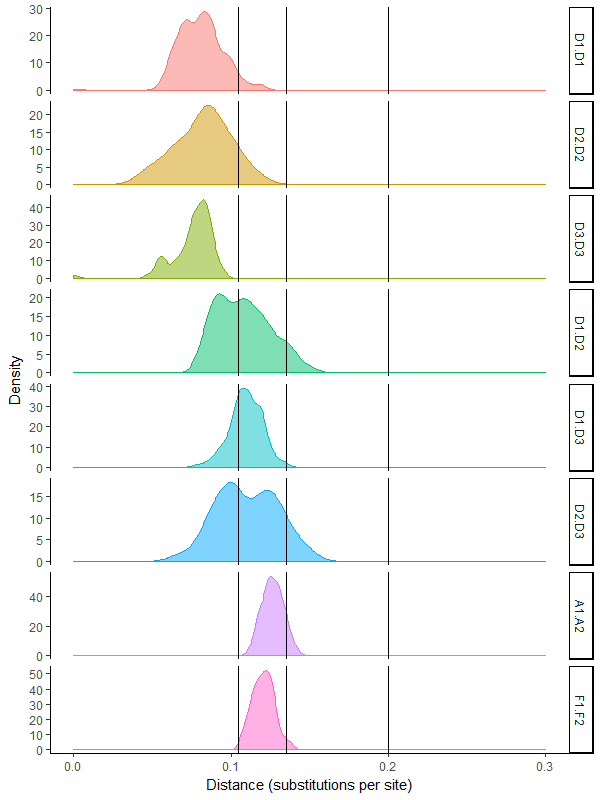


Fig. S5. Genetic distance comparisons between HIV-1 groups, subtypes and sub-subtypes using *pol* sequences. X-axis scale lines indicate genetic distance thresholds allowing, in our alignment and model conditions, for group, subtype and sub-subtype identification. Genetic distance ranges compatible with intra-sub-subtype, inter-sub-subtype, inter-subtype and inter-group comparisons are indicated by the numbers 1, 2, 3 and 4, respectively.

**
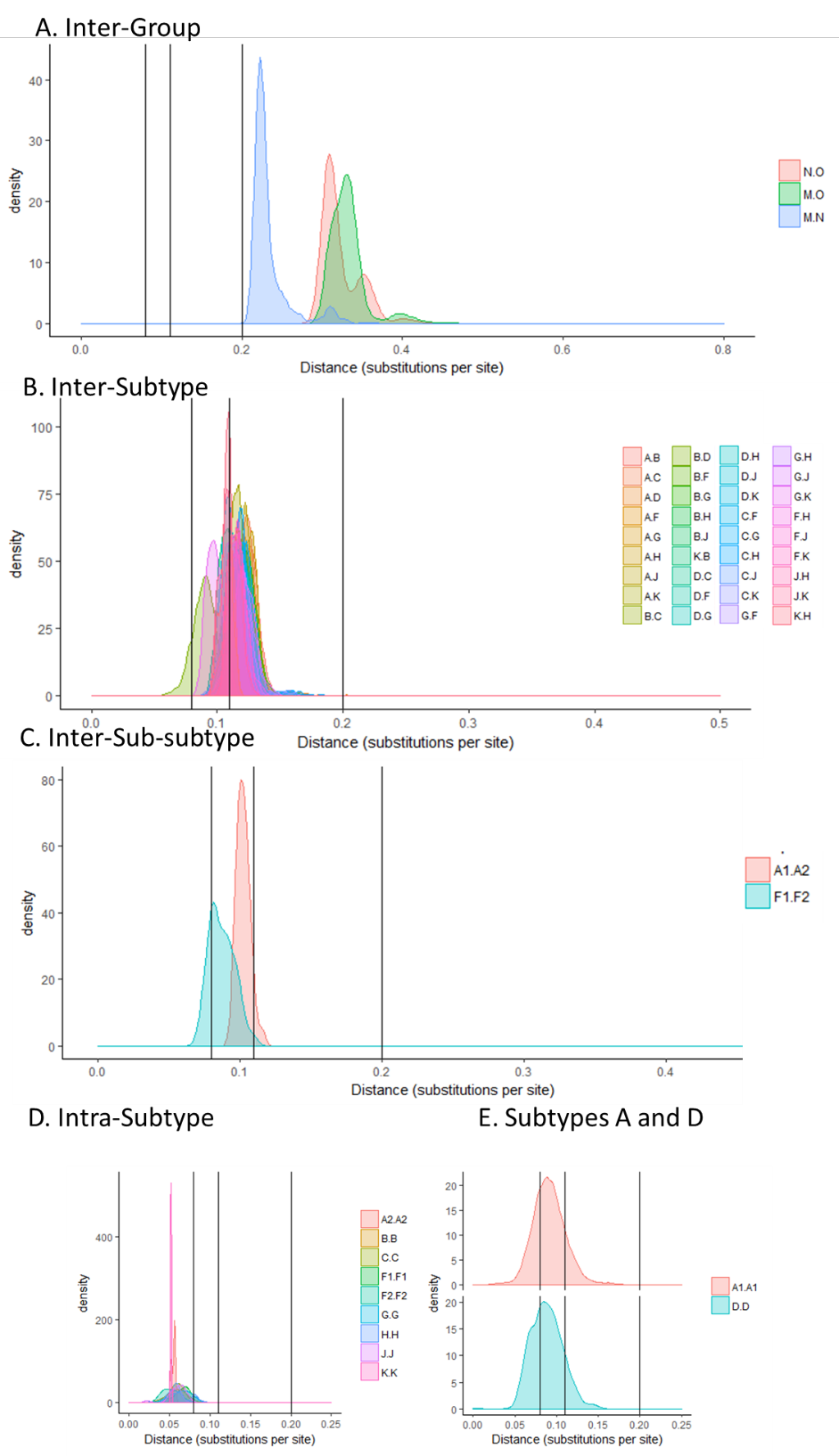
**

**Fig. S6. Subtypes A (A) and D (B) *pol* sequences maximum likelihood phylogenetic trees.** Names of all sub-subtypes are indicated according to the new classification proposal.


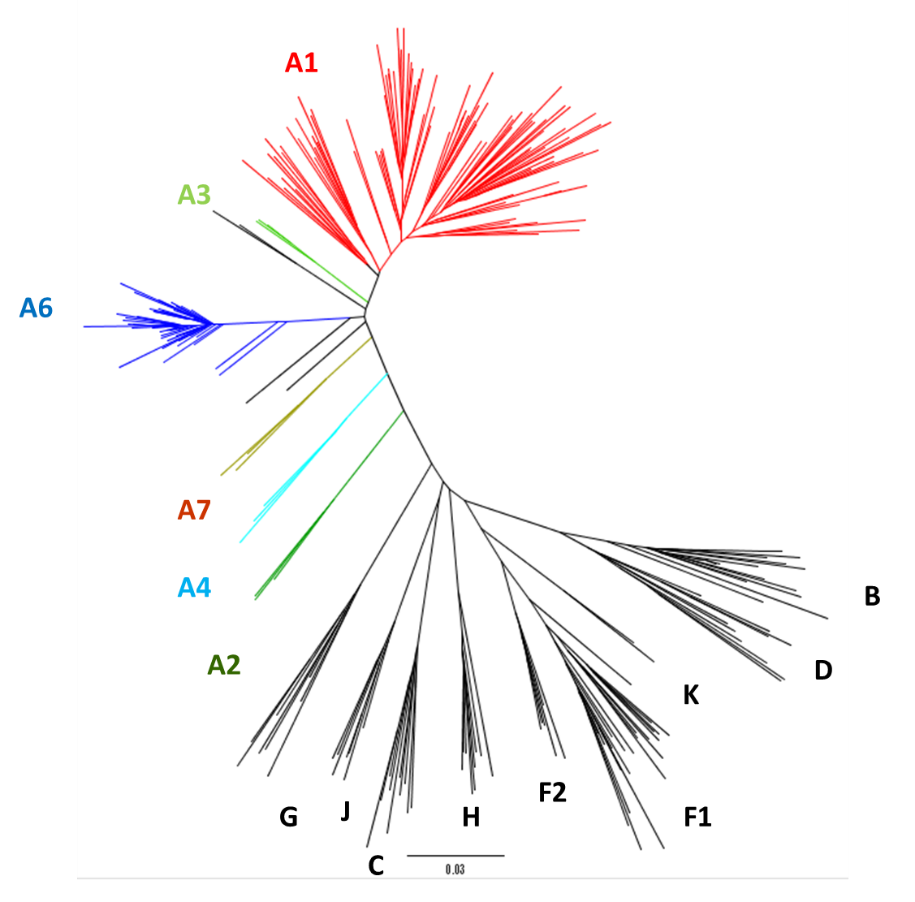


**A.**


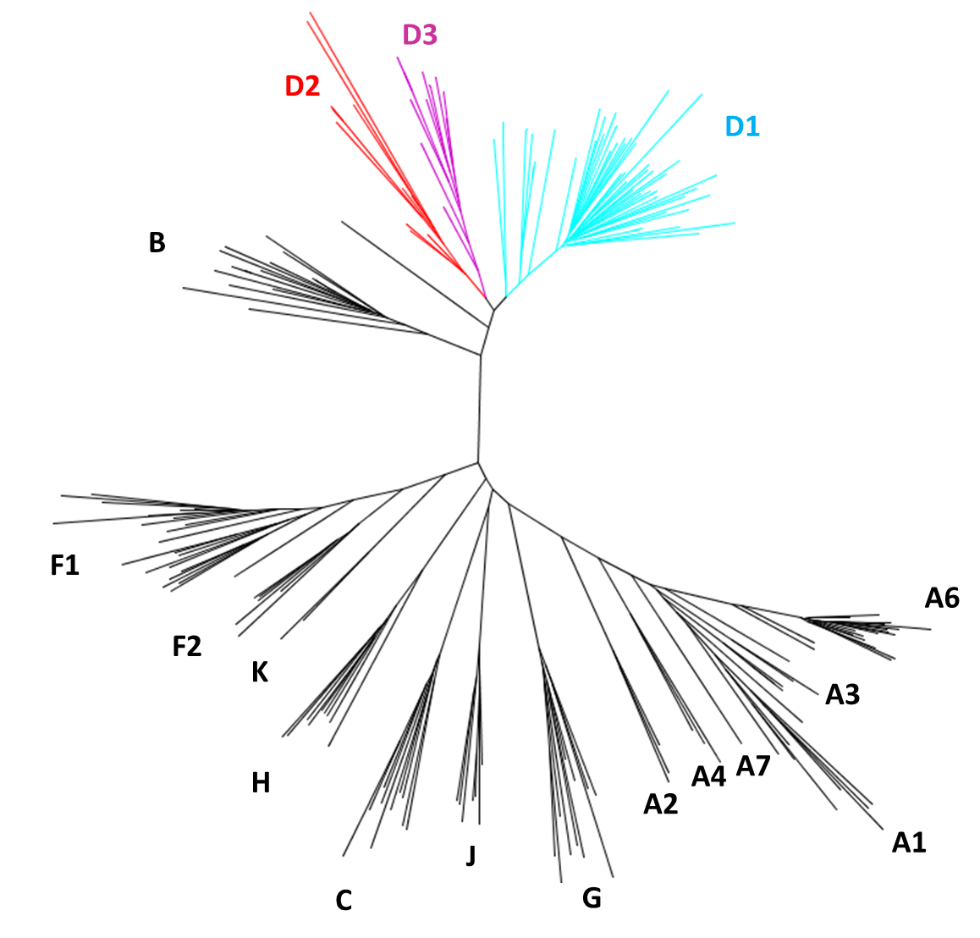


**B.**

Fig. S7. *Pol* gene genetic distance comparisons between HIV-1 subtype A sub-subtypes according to our classification proposal. X-axis scale lines indicate genetic distance thresholds allowing, in our alignment and model conditions, for group, subtype and sub-subtype identification.


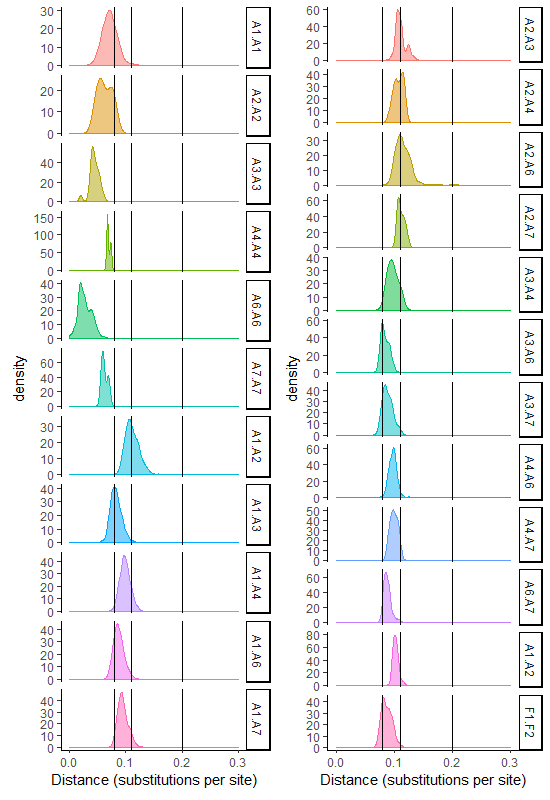


**Fig. S8. *Pol* gene genetic distance comparisons between HIV-1 subtype D sub-subtypes according to our classification proposal.** X-axis scale lines indicate genetic distance thresholds allowing, in our alignment and model conditions, for group, subtype and sub-subtype identification.


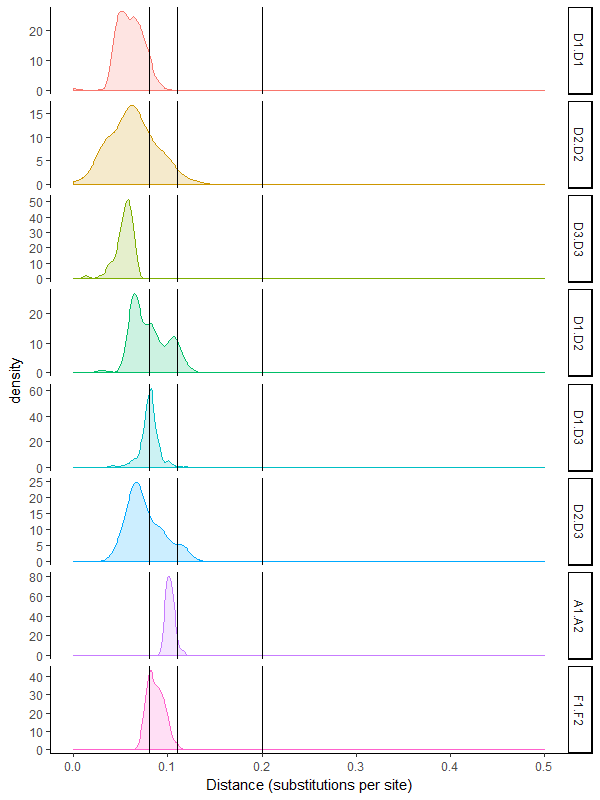


Table S1. Full genome sequence used for our analysis and the corresponding clade in our classification proposal.

**Subtype A**

| Accession number | Clade | Country | Years |
| --- | --- | --- | --- |
| AB098332 | A1 | UG | 1992 |
| AB098333 | A1 | UG | 1992 |
| AB253421 | A1 | RW | 1992 |
| AB287377 | A1 | RW | 1992 |
| AB287378 | A1 | RW | 1993 |
| AB485632 | A1 | UG | - |
| AF004885 | A1 | KE | 1994 |
| AF069669 | A1 | SE | 1995 |
| AF069670 | A1 | SE | 1994 |
| AF069671 | A1 | SE | 1994 |
| AF069673 | A1 | SE | 1995 |
| AF107771 | A1 | SE | 1995 |
| AF361872 | A1 | TZ | 1997 |
| AF361873 | A1 | TZ | 1997 |
| AF457052 | A1 | KE | 2000 |
| AF457053 | A1 | KE | 2000 |
| AF457055 | A1 | KE | 2000 |
| AF457063 | A1 | KE | 1999 |
| AF457065 | A1 | KE | 1999 |
| AF457066 | A1 | KE | 2000 |
| AF457067 | A1 | KE | 2000 |
| AF457068 | A1 | KE | 2000 |
| AF457069 | A1 | KE | 2000 |
| AF457070 | A1 | KE | 2000 |
| AF457075 | A1 | KE | 1999 |
| AF457077 | A1 | KE | 2000 |
| AF457079 | A1 | KE | 2000 |
| AF457080 | A1 | KE | 2000 |
| AF457081 | A1 | KE | 2000 |
| AF457083 | A1 | KE | 2000 |
| AF457084 | A1 | KE | 2000 |
| AF457086 | A1 | KE | 2000 |
| AF457089 | A1 | KE | 2000 |
| AF484478 | A1 | UG | 1999 |
| AF484493 | A1 | UG | 1999 |
| AF484507 | A1 | UG | 1998 |
| AF484508 | A1 | UG | 1998 |
| AF484509 | A1 | UG | 1998 |
| AF484512 | A1 | UG | 1998 |
| AF539405 | A1 | KE | 1986 |
| AY253305 | A1 | TZ | 2001 |
| AY253314 | A1 | TZ | 2001 |
| AY322184 | A1 | KE | 1986 |
| AY322185 | A1 | KE | 1997 |
| AY322190 | A1 | KE | 1997 |
| AY322193 | A1 | KE | 1997 |
| AY713406 | A1 | RW | 1993 |
| AY713407 | A1 | UG | 1992 |
| DQ676873 | A1 | AU | 2004 |
| EU110085 | A1 | KE | 2001 |
| EU110087 | A1 | KE | 2001 |
| EU110088 | A1 | KE | 2001 |
| EU110092 | A1 | KE | 2002 |
| EU110094 | A1 | KE | 2002 |
| FJ388893 | A1 | CY | 2005 |
| FJ388894 | A1 | CY | 2005 |
| FJ388903 | A1 | CY | 2005 |
| FJ388909 | A1 | CY | 2005 |
| FJ388925 | A1 | CY | 2005 |
| FJ388932 | A1 | CY | 2005 |
| FJ388938 | A1 | CY | 2005 |
| FJ388942 | A1 | CY | 2005 |
| FJ623475 | A1 | KE | 2006 |
| FJ623476 | A1 | KE | 2006 |
| FJ623477 | A1 | KE | 2006 |
| FJ623478 | A1 | KE | 2006 |
| FJ623479 | A1 | KE | 2006 |
| FJ623480 | A1 | KE | 2006 |
| FJ623481 | A1 | KE | 2006 |
| FJ623482 | A1 | KE | 2006 |
| FJ623483 | A1 | KE | 2006 |
| FJ623485 | A1 | KE | 2006 |
| FJ623486 | A1 | KE | 2006 |
| FJ623487 | A1 | KE | 2006 |
| FJ623488 | A1 | KE | 2006 |
| FJ670519 | A1 | ES | 2005 |
| JF683748 | A1 | CY | 2007 |
| JF683759 | A1 | CY | 2007 |
| JF683760 | A1 | CY | 2007 |
| JF683761 | A1 | CY | 2007 |
| JF683767 | A1 | CY | 2008 |
| JF683779 | A1 | CY | 2008 |
| JF683782 | A1 | CY | 2008 |
| JF683783 | A1 | CY | 2008 |
| JX140650 | A1 | ES | 2005 |
| JX140651 | A1 | ES | 2006 |
| JX236669 | A1 | UG | 2007 |
| JX236671 | A1 | UG | 2007 |
| JX236676 | A1 | UG | 2007 |
| KF716472 | A1 | RW | 2011 |
| KF716474 | A1 | KE | 2011 |
| KF716475 | A1 | KE | 2011 |
| KF716478 | A1 | UG | 2009 |
| KF716486 | A1 | UG | 2011 |
| KF716499 | A1 | RW | 2003 |
| KF859745 | A1 | UG | 2011 |
| KP109490 | A1 | UG | 2009 |
| KP109528 | A1 | RW | 2007 |
| KP718918 | A1 | CM | 2007 |
| KP718928 | A1 | CM | 2008 |
| KT022360 | A1 | KE | 2004 |
| KT022361 | A1 | KE | 2004 |
| KT022363 | A1 | KE | 2004 |
| KT022364 | A1 | KE | 2004 |
| KT022365 | A1 | KE | 2004 |
| KT022367 | A1 | KE | 2004 |
| KT022368 | A1 | KE | 2004 |
| KT022369 | A1 | KE | 2004 |
| KT022370 | A1 | KE | 2005 |
| KT022372 | A1 | KE | 2005 |
| KT022373 | A1 | KE | 2005 |
| KT022374 | A1 | KE | 2005 |
| KT022375 | A1 | KE | 2005 |
| KT022376 | A1 | KE | 2005 |
| KT022377 | A1 | KE | 2005 |
| KT022378 | A1 | KE | 2006 |
| KT022380 | A1 | KE | 2006 |
| KT022381 | A1 | KE | 2006 |
| KT022382 | A1 | KE | 2006 |
| KT022383 | A1 | KE | 2006 |
| KT022384 | A1 | KE | 2006 |
| KT152839 | A1 | IN | 2009 |
| KT152840 | A1 | IN | 1997 |
| KT152841 | A1 | IN | 1999 |
| KT152842 | A1 | IN | 1999 |
| KT152843 | A1 | IN | 1999 |
| KT152846 | A1 | IN | 2000 |
| KT183312 | A1 | ZA | 2004 |
| KU168305 | A1 | CM | 2003 |
| KX228810 | A1 | UG | 2010 |
| KX228815 | A1 | TZ | 2005 |
| U51190 | A1 | UG | 1992 |
| AF286237 | A2 | CY | 1994 |
| AF286238 | A2 | CD | 1997 |
| GU201516 | A2 | CM | 2001 |
| AY521629 | A3 | SN | 2001 |
| AY521630 | A3 | SN | 1996 |
| AY521631 | A3 | SN | 2001 |
| DQ396400 | A3 | ZA | 2004 |
| JQ403028 | A3 | CH | 2003 |
| KJ948658 | A3 | ZA | 2000 |
| KU168256 | A3 | CD | 2002 |
| M62320 | A3 | UG | 1985 |
| AM000053 | A4 | CD | 1997 |
| AM000054 | A4 | CD | 1997 |
| AM000055 | A4 | CD | 2002 |
| AF193275 | A6 | BY | 1997 |
| AF413987 | A6 | UA | 2000 |
| AY500393 | A6 | RU | 2003 |
| AY829203 | A6 | UZ | 2002 |
| AY829205 | A6 | UZ | 2002 |
| AY829206 | A6 | UZ | 2002 |
| AY829208 | A6 | UZ | 2002 |
| AY829209 | A6 | UZ | 2002 |
| AY829210 | A6 | UZ | 2002 |
| AY829212 | A6 | UZ | 2002 |
| DQ207944 | A6 | GE | 1999 |
| DQ823356 | A6 | UA | 2001 |
| DQ823357 | A6 | UA | 2001 |
| DQ823358 | A6 | UA | 2001 |
| DQ823359 | A6 | UA | 2001 |
| DQ823360 | A6 | UA | 2001 |
| DQ823361 | A6 | UA | 2001 |
| DQ823365 | A6 | UA | 2001 |
| DQ823366 | A6 | UA | 2001 |
| DQ823367 | A6 | UA | 2001 |
| EF545108 | A6 | RU | 2000 |
| EF589039 | A6 | KZ | 2002 |
| EF589040 | A6 | KZ | 2002 |
| EF589041 | A6 | KZ | 2002 |
| EF589042 | A6 | KZ | 2002 |
| EF589043 | A6 | KZ | 2002 |
| EF589044 | A6 | KZ | 2002 |
| EU861977 | A6 | IT | 2002 |
| FJ388892 | A6 | CY | 2005 |
| FJ388906 | A6 | CY | 2005 |
| FJ388950 | A6 | CY | 2006 |
| FJ388951 | A6 | CY | 2006 |
| FJ864679 | A6 | RU | 2008 |
| JF683763 | A6 | CY | 2007 |
| JF683780 | A6 | CY | 2008 |
| JF683798 | A6 | CY | 2009 |
| JQ292891 | A6 | RU | 2007 |
| JQ292892 | A6 | RU | 2002 |
| JQ292893 | A6 | RU | 2008 |
| JQ292894 | A6 | RU | 2008 |
| JQ292895 | A6 | RU | 2005 |
| JQ292896 | A6 | RU | 2006 |
| JQ292897 | A6 | RU | 2006 |
| JQ292898 | A6 | RU | 2006 |
| JQ292899 | A6 | RU | 2006 |
| JQ292900 | A6 | RU | 2006 |
| JX500694 | A6 | RU | 2011 |
| JX500695 | A6 | RU | 2010 |
| JX500696 | A6 | RU | 2010 |
| KF716491 | A6 | RU | 2008 |
| KF716492 | A6 | RU | 2008 |
| KT983615 | A6 | BY | 2013 |
| KX389608 | A7 | NG | 2010 |
| KX389622 | A7 | NG | 2009 |

**Subtype B**

| Accession number | Clade | Country | Year |
| --- | --- | --- | --- |
| X01762 | B | FR | 1983 |
| U71182 | B | CN | - |
| U69593 | B | US | 1990 |
| U69591 | B | US | 1990 |
| U69590 | B | US | 1990 |
| U69589 | B | US | 1990 |
| U69588 | B | US | 1985 |
| U69586 | B | US | 1985 |
| U69585 | B | US | 1985 |
| U69584 | B | US | 1985 |
| U63632 | B | US | 1986 |
| U43141 | B | DE | 1986 |
| U43096 | B | DE | 1986 |
| U39362 | B | US | 1989 |
| U34604 | B | NL | 1986 |
| U34603 | B | NL | 1986 |
| U26942 | B | FR | 1985 |
| U26546 | B | US | 1988 |
| U23487 | B | GB | - |
| U21135 | B | US | 1990 |
| AF049495 | B | US | - |
| AY779557 | B | CA | 1998 |
| AY779559 | B | CA | 1992 |
| AY779560 | B | CA | 1994 |
| AY779561 | B | CA | 1992 |
| AY779562 | B | CA | 1993 |
| AY779563 | B | CA | 1996 |
| FJ469752 | B | US | - |
| M17449 | B | US | 1984 |
| NC_001802 | B | FR | 1983 |
| U23487 | B | GB | - |
| M93259 | B | US | 1986 |
| M93258 | B | US | 1986 |
| M38429 | B | US | 1986 |
| M26727 | B | GA | 1988 |
| M17451 | B | US | 1983 |
| M17449 | B | US | 1984 |
| M15654 | B | FR | 1983 |
| L31963 | B | FR | 1983 |
| L02317 | B | US | 1987 |
| KJ948660 | B | ZA | 2002 |
| KJ948657 | B | ZA | 2000 |
| KJ849820 | B | BR | 2010 |
| KJ849818 | B | BR | 2010 |
| KJ849817 | B | BR | 2010 |
| KJ849815 | B | BR | 2010 |
| KJ849814 | B | BR | 2010 |
| KJ849812 | B | BR | 2010 |
| KJ849811 | B | BR | 2010 |
| KJ849808 | B | BR | 2010 |
| KJ849807 | B | BR | 2010 |
| KJ849805 | B | BR | 2010 |
| KJ849804 | B | BR | 2010 |
| KJ849803 | B | BR | 2010 |
| KJ849801 | B | BR | 2010 |
| KJ849799 | B | BR | 2010 |
| KJ849796 | B | BR | 2010 |
| KJ849790 | B | BR | 2010 |
| KJ849786 | B | BR | 2010 |
| KJ849785 | B | BR | 2010 |
| KJ849784 | B | BR | 2010 |
| KJ849780 | B | BR | 2010 |
| KJ849767 | B | BR | 2010 |
| KJ769147 | B | TH | 2006 |
| KJ140267 | B | KR | 1992 |
| KJ140266 | B | KR | 1992 |
| KJ140265 | B | KR | 2002 |
| KJ140264 | B | KR | 2002 |
| KJ140263 | B | KR | 2007 |
| KJ140262 | B | KR | 1993 |
| KJ140261 | B | KR | 1992 |
| KJ140260 | B | KR | 2003 |
| KJ140258 | B | KR | 1992 |
| KJ140257 | B | KR | 1992 |
| KJ140256 | B | KR | 1992 |
| KJ140255 | B | KR | 1992 |
| KJ140254 | B | KR | 2002 |
| KJ140253 | B | KR | 1993 |
| KJ140252 | B | KR | 1992 |
| KJ140251 | B | KR | 1992 |
| KJ140250 | B | KR | 1995 |
| KJ140249 | B | KR | 2002 |
| KJ140248 | B | KR | 1992 |
| KJ140247 | B | KR | 1992 |
| KJ019215 | B | GB | 1994 |
| KF716494 | B | FR | 2009 |
| KF561443 | B | KR | 1993 |
| KF561442 | B | KR | 1991 |
| KF561441 | B | KR | 2012 |
| KF561440 | B | KR | 2012 |
| KF526318 | B | US | 2011 |
| KF526312 | B | US | 2011 |
| KF526298 | B | US | 2011 |
| KF526287 | B | US | 2011 |
| KF526280 | B | US | 2011 |
| KF526279 | B | US | 2011 |
| KF526276 | B | US | 2011 |
| KF526270 | B | US | 2011 |
| KF526265 | B | US | 2011 |
| KF526236 | B | US | 2011 |
| KF526231 | B | US | 2011 |
| KF526204 | B | US | 2011 |
| KF526203 | B | US | 2011 |
| KF526202 | B | US | 2011 |
| KF526201 | B | US | 2011 |
| KF526192 | B | US | 2011 |
| KF526179 | B | US | 2011 |
| KF526176 | B | US | 2011 |
| KF526174 | B | US | 2011 |
| KF526120 | B | US | 2010 |
| KF384814 | B | US | 2011 |
| KF384813 | B | US | 2011 |
| KF384811 | B | US | 2011 |
| KF384810 | B | US | 2011 |
| KF384809 | B | US | 2011 |
| KF384808 | B | US | 2011 |
| KF384807 | B | US | 2011 |
| KF384806 | B | US | 2011 |
| KF384805 | B | US | 2011 |
| KF384804 | B | US | 2011 |
| KF384803 | B | US | 2011 |
| KF384802 | B | US | 2011 |
| KF384801 | B | US | 2011 |
| KF384800 | B | US | 2011 |
| KF384799 | B | US | 2011 |
| KF384798 | B | US | 2011 |
| KC935959 | B | US | 2009 |
| KC935958 | B | US | 2009 |
| KC899011 | B | CN | 2009 |
| KC797229 | B | CH | 2008 |
| KC797225 | B | CH | 2008 |
| KC797224 | B | CH | 2008 |
| KC797223 | B | CH | 2008 |
| KC797178 | B | CH | 2000 |
| KC797177 | B | CH | 2000 |
| KC797175 | B | CH | 2000 |
| KC797174 | B | CH | 2000 |
| KC797171 | B | CH | 2000 |
| KC596069 | B | PL | - |
| KC596067 | B | DE | - |
| KC596066 | B | CN | 2009 |
| KC473846 | B | US | 2003 |
| KC473843 | B | ES | 2010 |
| KC473842 | B | ES | 2010 |
| KC473841 | B | ES | 2009 |
| KC473835 | B | US | 2011 |
| KC473834 | B | US | 2011 |
| KC473833 | B | US | 2011 |
| KC473832 | B | US | 2011 |
| KC473831 | B | US | 2011 |
| KC473830 | B | US | 2010 |
| KC473829 | B | US | 2010 |
| KC473828 | B | US | 2010 |
| KC473827 | B | US | 2010 |
| KC473826 | B | US | 2010 |
| KC473825 | B | US | 2010 |
| KC473824 | B | US | 2009 |
| K03455 | B | FR | 1983 |
| K02083 | B | FR | 1983 |
| K02013 | B | FR | 1983 |
| K02007 | B | US | 1983 |
| JX974235 | B | US | 2008 |
| JX960599 | B | CN | 2009 |
| JX960598 | B | CN | 2009 |
| JX960597 | B | CN | 2009 |
| JX863924 | B | US | 2009 |
| JX863923 | B | US | 2009 |
| JX863922 | B | US | 2007 |
| JX863921 | B | US | 2007 |
| JX863919 | B | US | 2005 |
| JX503073 | B | FR | 1985 |
| JX500709 | B | RU | 2009 |
| JX500708 | B | RU | 2011 |
| JX500707 | B | RU | 2010 |
| JX447165 | B | TH | 2007 |
| JX447161 | B | TH | 2007 |
| JX447158 | B | TH | 2007 |
| JX447157 | B | TH | 2007 |
| JX446805 | B | TH | 2006 |
| JX446801 | B | TH | 2006 |
| JX446797 | B | TH | 2006 |
| JX446795 | B | TH | 2006 |
| JX140659 | B | ES | 2010 |
| JX140658 | B | CN | 2010 |
| JX140657 | B | US | 2009 |
| JX140656 | B | ES | 2009 |
| JX140655 | B | ES | 2008 |
| JX140654 | B | FR | 2008 |
| JX140653 | B | ES | 2008 |
| JX140652 | B | FR | 2005 |
| JQ416162 | B | DE | 2005 |
| JQ416161 | B | DE | 2004 |
| JQ416159 | B | DE | 2004 |
| JQ403107 | B | US | 2004 |
| JQ403105 | B | US | 2002 |
| JQ403060 | B | US | 2007 |
| JQ403059 | B | US | 2007 |
| JQ403056 | B | US | 2007 |
| JQ403055 | B | DE | 2008 |
| JQ403049 | B | DE | 2005 |
| JQ403048 | B | DE | 2003 |
| JQ403045 | B | CH | 2003 |
| JQ403042 | B | CH | 2004 |
| JQ403040 | B | FR | 1985 |
| JQ403039 | B | DE | 2004 |
| JQ403038 | B | DE | 2004 |
| JQ403037 | B | DE | 2004 |
| JQ403036 | B | US | 2008 |
| JQ403035 | B | US | 2008 |
| JQ403033 | B | US | 2008 |
| JQ403029 | B | CH | 2002 |
| JQ403026 | B | CH | 2001 |
| JQ403025 | B | CH | 2001 |
| JQ403024 | B | CH | 2001 |
| JQ403023 | B | CH | 2000 |
| JQ403022 | B | CH | 2000 |
| JQ403021 | B | CH | 2002 |
| JQ403019 | B | CH | 2002 |
| JQ341411 | B | KR | 2007 |
| JQ316135 | B | KR | 2003 |
| JQ316134 | B | KR | 2005 |
| JQ316133 | B | KR | 2004 |
| JQ316132 | B | KR | 2003 |
| JQ316131 | B | KR | 2003 |
| JQ316130 | B | KR | 2004 |
| JQ316129 | B | KR | 1999 |
| JQ316128 | B | KR | 2003 |
| JQ316127 | B | KR | 2003 |
| JQ316126 | B | KR | 2004 |
| JN944946 | B | US | 2000 |
| JN944942 | B | US | 2006 |
| JN944941 | B | US | 2006 |
| JN944940 | B | US | 2006 |
| JN944939 | B | US | 2006 |
| JN944938 | B | US | 2000 |
| JN944936 | B | US | 2001 |
| JN944930 | B | US | 2000 |
| JN944928 | B | US | 1991 |
| JN944918 | B | US | 2000 |
| JN944912 | B | US | 2001 |
| JN944909 | B | US | 2006 |
| JN944907 | B | US | 2006 |
| JN944905 | B | US | 2006 |
| JN944897 | B | US | 2006 |
| JN860769 | B | TH | 2008 |
| JN692480 | B | BR | 2006 |
| JN692479 | B | BR | 2006 |
| JN692476 | B | BR | 2005 |
| JN692475 | B | BR | 2005 |
| JN692474 | B | BR | 2005 |
| JN692473 | B | BR | 2005 |
| JN692472 | B | BR | 2005 |
| JN692471 | B | BR | 2005 |
| JN692470 | B | BR | 2002 |
| JN692468 | B | BR | 2005 |
| JN692467 | B | BR | 2005 |
| JN692466 | B | BR | 2005 |
| JN692465 | B | BR | 2005 |
| JN692464 | B | BR | 2005 |
| JN692463 | B | BR | 2005 |
| JN692462 | B | BR | 2005 |
| JN692461 | B | BR | 2005 |
| JN692460 | B | BR | 2005 |
| JN692458 | B | BR | 2004 |
| JN692457 | B | BR | 2004 |
| JN692455 | B | BR | 2004 |
| JN692453 | B | BR | 2004 |
| JN692452 | B | BR | 2004 |
| JN692451 | B | BR | 2004 |
| JN692450 | B | BR | 2004 |
| JN692447 | B | BR | 2003 |
| JN692446 | B | BR | 2003 |
| JN692445 | B | BR | 2003 |
| JN692444 | B | BR | 2002 |
| JN692443 | B | BR | 2002 |
| JN692439 | B | BR | 2002 |
| JN692436 | B | BR | 2002 |
| JN692435 | B | BR | 2002 |
| JN692433 | B | BR | 2002 |
| JN692432 | B | BR | 2002 |
| JN692431 | B | BR | 2002 |
| JN397365 | B | US | 2010 |
| JN397364 | B | US | 2010 |
| JN397363 | B | US | 2011 |
| JN397362 | B | US | 2011 |
| JN251906 | B | PY | 2003 |
| JN251901 | B | PY | 2002 |
| JN251896 | B | PY | 2002 |
| JN248354 | B | TH | 2005 |
| JN248353 | B | TH | 2005 |
| JN248348 | B | TH | 2005 |
| JN248347 | B | TH | 2005 |
| JN248346 | B | TH | 2005 |
| JN248345 | B | TH | 2005 |
| JN248344 | B | TH | 2005 |
| JN248343 | B | TH | 2005 |
| JN248337 | B | TH | 2004 |
| JN248335 | B | TH | 2004 |
| JN248333 | B | TH | 2004 |
| JN248329 | B | TH | 2004 |
| JN248321 | B | TH | 2004 |
| JN235965 | B | UY | 1999 |
| JN235959 | B | UY | 1999 |
| JN235958 | B | UY | 2002 |
| JN024572 | B | US | 2005 |
| JN024571 | B | US | 2005 |
| JN024559 | B | US | 2005 |
| JN024557 | B | US | 2005 |
| JN024554 | B | US | 2005 |
| JN024544 | B | US | 2005 |
| JN024538 | B | US | 2005 |
| JN024535 | B | US | 2005 |
| JN024534 | B | US | 2005 |
| JN024532 | B | US | 2005 |
| JN024528 | B | US | 2005 |
| JN024542 | B | US | 2005 |
| JN024525 | B | US | 2005 |
| JN024522 | B | US | 2005 |
| JN024512 | B | US | 2004 |
| JN024509 | B | US | 2005 |
| JN024506 | B | US | 2005 |
| JN024502 | B | US | 1996 |
| JN024500 | B | US | 1996 |
| JN024499 | B | US | 1996 |
| JN024497 | B | US | 2004 |
| JN024495 | B | US | 2005 |
| JN024491 | B | US | 2005 |
| JN024468 | B | US | 2005 |
| JN024458 | B | US | 2005 |
| JN024457 | B | US | 2005 |
| JN024455 | B | US | 2005 |
| JN024454 | B | US | 2005 |
| JN024452 | B | US | 2005 |
| JN024445 | B | US | 2005 |
| JN024435 | B | US | 1996 |
| JN024432 | B | US | 1996 |
| JN024430 | B | US | 1996 |
| JN024428 | B | US | 1996 |
| JN024426 | B | US | 1996 |
| JN024424 | B | US | 1996 |
| JN024422 | B | US | 1996 |
| JN024420 | B | US | 2005 |
| JN024412 | B | US | 2005 |
| JN024399 | B | US | 2005 |
| JN024376 | B | US | 2005 |
| JN024366 | B | US | 2005 |
| JN024350 | B | US | 2005 |
| JN024345 | B | US | 2005 |
| JN024331 | B | US | 1996 |
| JN024329 | B | US | 1995 |
| JN024315 | B | US | 1995 |
| JN024306 | B | US | 1995 |
| JN024301 | B | US | 1995 |
| JN024275 | B | US | 1995 |
| JN024290 | B | US | 1995 |
| JN024274 | B | US | 1995 |
| JN024254 | B | US | 2004 |
| JN024236 | B | US | 2004 |
| JN024222 | B | US | 2004 |
| JN024204 | B | US | 2004 |
| JN024200 | B | US | 2005 |
| JN024196 | B | US | 2005 |
| JN024193 | B | US | 2005 |
| JN024191 | B | US | 2005 |
| JN024189 | B | US | 2005 |
| JN024186 | B | US | 2005 |
| JN024181 | B | US | 2005 |
| JN024179 | B | US | 2005 |
| JN024176 | B | US | 2005 |
| JN024166 | B | US | 2005 |
| JN024165 | B | US | 2005 |
| JN024164 | B | US | 2005 |
| JN024163 | B | US | 2005 |
| JN024161 | B | US | 2005 |
| JN024160 | B | US | 2005 |
| JN024158 | B | US | 2005 |
| JN024157 | B | US | 2005 |
| JN024156 | B | US | 2005 |
| JN024146 | B | US | 2005 |
| JN024145 | B | US | 2005 |
| JN024144 | B | US | 2005 |
| JN024143 | B | US | 2005 |
| JN024142 | B | US | 2005 |
| JN024141 | B | US | 2005 |
| JN024140 | B | US | 2005 |
| JN024139 | B | US | 2005 |
| JN024138 | B | US | 2005 |
| JN024137 | B | US | 2005 |
| JN024136 | B | US | 2005 |
| JN024123 | B | US | 2005 |
| JN024101 | B | US | 2004 |
| JF932500 | B | CN | 2007 |
| JF932499 | B | CN | 2007 |
| JF932498 | B | CN | 2008 |
| JF932497 | B | CN | 2009 |
| JF932496 | B | CN | 1998 |
| JF932495 | B | CN | 1998 |
| JF932494 | B | CN | 2009 |
| JF932493 | B | CN | 2007 |
| JF932492 | B | CN | 2003 |
| JF932491 | B | CN | 2007 |
| JF932490 | B | CN | 2007 |
| JF932489 | B | CN | 2007 |
| JF932488 | B | CN | 2007 |
| JF932487 | B | CN | 2007 |
| JF932486 | B | CN | 2007 |
| JF932485 | B | CN | 2007 |
| JF932484 | B | CN | 2007 |
| JF932483 | B | CN | 2007 |
| JF932482 | B | CN | 2006 |
| JF932481 | B | CN | 2008 |
| JF932480 | B | CN | 2008 |
| JF932479 | B | CN | 2008 |
| JF932478 | B | CN | 2008 |
| JF932477 | B | CN | 2007 |
| JF932476 | B | CN | 2007 |
| JF932475 | B | CN | 2007 |
| JF932474 | B | CN | 2007 |
| JF932473 | B | CN | 2007 |
| JF932472 | B | CN | 2007 |
| JF932471 | B | CN | 2007 |
| JF932470 | B | CN | 2007 |
| JF932469 | B | CN | 2007 |
| JF932468 | B | CN | 2007 |
| JF689897 | B | US | 2008 |
| JF689896 | B | US | 2008 |
| JF689895 | B | US | 2008 |
| JF689894 | B | US | 2008 |
| JF689892 | B | US | 2007 |
| JF689890 | B | US | 2007 |
| JF689889 | B | US | 2007 |
| JF689887 | B | US | 2007 |
| JF689886 | B | US | 2007 |
| JF689885 | B | US | 2007 |
| JF689884 | B | US | 2007 |
| JF689883 | B | US | 2007 |
| JF689879 | B | US | 2007 |
| JF689877 | B | US | 2007 |
| JF689876 | B | US | 2006 |
| JF689875 | B | US | 2006 |
| JF689874 | B | US | 2006 |
| JF689873 | B | US | 2006 |
| JF689872 | B | US | 2006 |
| JF689871 | B | US | 2006 |
| JF689870 | B | US | 2006 |
| JF689868 | B | US | 2006 |
| JF689867 | B | US | 2006 |
| JF689866 | B | US | 2006 |
| JF689865 | B | US | 2006 |
| JF689864 | B | US | 2006 |
| JF689863 | B | US | 2006 |
| JF689862 | B | US | 2006 |
| JF689860 | B | US | 2005 |
| JF689859 | B | US | 2005 |
| JF689857 | B | US | 2005 |
| JF689856 | B | US | 2005 |
| JF689854 | B | US | 2005 |
| JF689852 | B | US | 2005 |
| JF683809 | B | CY | 2009 |
| JF683808 | B | CY | 2009 |
| JF683807 | B | CY | 2009 |
| JF683805 | B | CY | 2009 |
| JF683804 | B | CY | 2009 |
| JF683801 | B | CY | 2009 |
| JF683797 | B | CY | 2009 |
| JF683796 | B | CY | 2009 |
| JF683794 | B | CY | 2009 |
| JF683793 | B | CY | 2009 |
| JF683791 | B | CY | 2009 |
| JF683790 | B | CY | 2009 |
| JF683788 | B | CY | 2009 |
| JF683787 | B | CY | 2009 |
| JF683785 | B | CY | 2008 |
| JF683784 | B | CY | 2008 |
| JF683781 | B | CY | 2008 |
| JF683778 | B | CY | 2008 |
| JF683775 | B | CY | 2008 |
| JF683773 | B | CY | 2008 |
| JF683769 | B | CY | 2008 |
| JF683765 | B | CY | 2007 |
| JF683764 | B | CY | 2007 |
| JF683756 | B | CY | 2007 |
| JF683754 | B | CY | 2007 |
| JF683753 | B | CY | 2007 |
| JF683751 | B | CY | 2007 |
| JF683749 | B | CY | 2007 |
| JF683747 | B | CY | 2007 |
| JF683746 | B | CY | 2007 |
| JF683743 | B | CY | 2007 |
| JF683742 | B | CY | 2007 |
| JF683741 | B | CY | 2007 |
| JF683738 | B | CY | 2007 |
| JF320629 | B | US | 2006 |
| JF320628 | B | US | 2006 |
| JF320627 | B | US | 2006 |
| JF320626 | B | US | 2006 |
| JF320624 | B | US | 2006 |
| JF320622 | B | US | 2006 |
| JF320621 | B | US | 2006 |
| JF320620 | B | US | 2006 |
| JF320619 | B | US | 2006 |
| JF320617 | B | US | 2006 |
| JF320616 | B | US | 2006 |
| JF320615 | B | US | 2006 |
| JF320613 | B | US | 2006 |
| JF320577 | B | US | 2007 |
| JF320564 | B | US | 2006 |
| JF320562 | B | US | 2007 |
| JF320536 | B | US | 2007 |
| JF320535 | B | US | 2007 |
| JF320534 | B | US | 2007 |
| JF320533 | B | US | 2007 |
| JF320532 | B | US | 2007 |
| JF320531 | B | US | 2007 |
| JF320530 | B | US | 2007 |
| JF320529 | B | US | 2006 |
| JF320526 | B | US | 2006 |
| JF320519 | B | US | 2006 |
| JF320518 | B | US | 2006 |
| JF320516 | B | US | 2006 |
| JF320515 | B | US | 2006 |
| JF320492 | B | US | 2005 |
| JF320491 | B | US | 2005 |
| JF320490 | B | US | 2005 |
| JF320489 | B | US | 2005 |
| JF320488 | B | US | 2005 |
| JF320487 | B | US | 2005 |
| JF320486 | B | US | 2005 |
| JF320485 | B | US | 2005 |
| JF320484 | B | US | 2005 |
| JF320483 | B | US | 2005 |
| JF320482 | B | US | 2005 |
| JF320478 | B | US | 2005 |
| JF320477 | B | US | 2005 |
| JF320470 | B | US | 2007 |
| JF320469 | B | US | 2007 |
| JF320468 | B | US | 2007 |
| JF320467 | B | US | 2007 |
| JF320466 | B | US | 2007 |
| JF320465 | B | US | 2007 |
| JF320464 | B | US | 2007 |
| JF320463 | B | US | 2007 |
| JF320462 | B | US | 2007 |
| JF320461 | B | US | 2007 |
| JF320429 | B | CA | 2006 |
| JF320422 | B | CA | 2007 |
| JF320411 | B | CA | 2006 |
| JF320374 | B | US | 2006 |
| JF320366 | B | US | 2006 |
| JF320365 | B | US | 2006 |
| JF320364 | B | US | 2006 |
| JF320363 | B | US | 2006 |
| JF320361 | B | US | 2005 |
| JF320356 | B | US | 2006 |
| JF320271 | B | US | 2006 |
| JF320269 | B | US | 2006 |
| JF320268 | B | US | 2006 |
| JF320267 | B | US | 2006 |
| JF320263 | B | US | 2006 |
| JF320257 | B | US | 2006 |
| JF320247 | B | US | 2006 |
| JF320244 | B | PE | 2006 |
| JF320242 | B | PE | 2006 |
| JF320241 | B | PE | 2006 |
| JF320225 | B | PE | 2007 |
| JF320223 | B | PE | 2006 |
| JF320220 | B | PE | 2007 |
| JF320219 | B | PE | 2006 |
| JF320218 | B | PE | 2006 |
| JF320215 | B | PE | 2006 |
| JF320214 | B | PE | 2006 |
| JF320209 | B | PE | 2006 |
| JF320207 | B | US | 2007 |
| JF320205 | B | US | 2007 |
| JF320203 | B | PE | 2006 |
| JF320202 | B | US | 2007 |
| JF320201 | B | PE | 2006 |
| JF320199 | B | PE | 2007 |
| JF320198 | B | PE | 2007 |
| JF320197 | B | US | 2007 |
| JF320196 | B | PE | 2006 |
| JF320195 | B | PE | 2007 |
| JF320193 | B | PE | 2006 |
| JF320192 | B | US | 2005 |
| JF320191 | B | PE | 2007 |
| JF320189 | B | PE | 2007 |
| JF320187 | B | PE | 2006 |
| JF320186 | B | PE | 2006 |
| JF320185 | B | US | 2005 |
| JF320183 | B | PE | 2006 |
| JF320182 | B | US | 2007 |
| JF320181 | B | US | 2007 |
| JF320179 | B | US | 2007 |
| JF320171 | B | US | 2006 |
| JF320169 | B | US | 2006 |
| JF320160 | B | US | 2006 |
| JF320158 | B | US | 2007 |
| JF320157 | B | US | 2007 |
| JF320156 | B | US | 2007 |
| JF320155 | B | US | 2007 |
| JF320154 | B | US | 2006 |
| JF320153 | B | US | 2006 |
| JF320152 | B | US | 2006 |
| JF320151 | B | US | 2006 |
| JF320150 | B | US | 2007 |
| JF320149 | B | US | 2006 |
| JF320148 | B | US | 2006 |
| JF320147 | B | US | 2006 |
| JF320146 | B | US | 2006 |
| JF320145 | B | US | 2006 |
| JF320144 | B | US | 2006 |
| JF320120 | B | US | 2006 |
| JF320097 | B | US | 2006 |
| JF320082 | B | US | 2006 |
| JF320081 | B | US | 2006 |
| JF320071 | B | US | 2006 |
| JF320070 | B | US | 2007 |
| JF320069 | B | US | 2007 |
| JF320068 | B | US | 2006 |
| JF320066 | B | US | 2005 |
| JF320065 | B | US | 2006 |
| JF320064 | B | US | 2006 |
| JF320062 | B | US | 2006 |
| JF320061 | B | US | 2005 |
| JF320060 | B | US | 2006 |
| JF320059 | B | US | 2005 |
| JF320058 | B | US | 2006 |
| JF320056 | B | US | 2006 |
| JF320055 | B | US | 2006 |
| JF320054 | B | US | 2005 |
| JF320053 | B | US | 2006 |
| JF320052 | B | US | 2007 |
| JF320051 | B | US | 2006 |
| JF320049 | B | US | 2006 |
| JF320048 | B | US | 2006 |
| JF320047 | B | US | 2006 |
| JF320045 | B | US | 2007 |
| JF320044 | B | US | 2006 |
| JF320036 | B | US | 2006 |
| JF320028 | B | PE | 2007 |
| JF320027 | B | PE | 2007 |
| JF320026 | B | PE | 2007 |
| JF320025 | B | PE | 2007 |
| JF320024 | B | PE | 2007 |
| JF320023 | B | PE | 2007 |
| JF320022 | B | PE | 2007 |
| JF320021 | B | PE | 2007 |
| JF320020 | B | PE | 2007 |
| JF320019 | B | PE | 2007 |
| JF320018 | B | PE | 2007 |
| JF320017 | B | PE | 2007 |
| JF320012 | B | PE | 2007 |
| JF320008 | B | PE | 2006 |
| JF320003 | B | US | 2006 |
| HQ215556 | B | CN | 2009 |
| HQ215554 | B | CN | 2008 |
| HM586212 | B | GB | 2005 |
| HM586204 | B | GB | 2005 |
| HM586202 | B | GB | 2005 |
| HM586200 | B | GB | 2004 |
| HM586198 | B | GB | 2004 |
| HM586193 | B | GB | 2004 |
| HM586187 | B | GB | 2003 |
| HM469983 | B | ES | 1989 |
| HM469981 | B | ES | 1989 |
| HM469980 | B | ES | 1989 |
| HM469979 | B | ES | 1989 |
| HM469978 | B | ES | 1989 |
| HM469977 | B | ES | 1989 |
| HM469976 | B | ES | 1989 |
| HM469975 | B | ES | 1989 |
| HM469972 | B | ES | 1989 |
| GU733713 | B | US | 2009 |
| GU647198 | B | CN | 1999 |
| GU647197 | B | CN | 1999 |
| GU647196 | B | CN | 1999 |
| GU362886 | B | ES | 2008 |
| GU362885 | B | ES | 2009 |
| GU362881 | B | ES | 2009 |
| GU177863 | B | CN | 1999 |
| GQ386795 | B | ES | 1989 |
| GQ386794 | B | ES | 1989 |
| GQ386791 | B | ES | 1989 |
| GQ386778 | B | ES | 1989 |
| GQ386775 | B | ES | 1989 |
| GQ372990 | B | ES | 2008 |
| GQ372988 | B | ES | 2008 |
| FJ853622 | B | ES | 2008 |
| FJ853620 | B | ES | 2008 |
| FJ694790 | B | DK | 2007 |
| FJ670531 | B | ES | 2008 |
| FJ670525 | B | ES | 2008 |
| FJ670524 | B | ES | 2008 |
| FJ647145 | B | ZA | 1985 |
| FJ496179 | B | US | 2000 |
| FJ496177 | B | US | 2000 |
| FJ496175 | B | US | 2000 |
| FJ496172 | B | US | 2000 |
| FJ496171 | B | US | 2000 |
| FJ496169 | B | US | 2000 |
| FJ496167 | B | US | 2000 |
| FJ496166 | B | US | 1990 |
| FJ496164 | B | US | 1990 |
| FJ496162 | B | US | 1990 |
| FJ496159 | B | US | 1990 |
| FJ496157 | B | US | 2001 |
| FJ496156 | B | US | 2001 |
| FJ496155 | B | US | 2001 |
| FJ496154 | B | US | 2001 |
| FJ496153 | B | US | 2001 |
| FJ496152 | B | US | 2001 |
| FJ496151 | B | US | 2001 |
| FJ496150 | B | US | 1991 |
| FJ496149 | B | US | 1991 |
| FJ496148 | B | US | 1991 |
| FJ496147 | B | US | 1991 |
| FJ496146 | B | US | 1991 |
| FJ496145 | B | US | 1991 |
| FJ496082 | B | US | 2005 |
| FJ496081 | B | US | 2005 |
| FJ496079 | B | US | 2002 |
| FJ496078 | B | US | 2002 |
| FJ496076 | B | US | 2002 |
| FJ496007 | B | US | 2006 |
| FJ496006 | B | US | 2006 |
| FJ496005 | B | US | 2006 |
| FJ496004 | B | US | 2006 |
| FJ496003 | B | US | 2006 |
| FJ496002 | B | US | 2006 |
| FJ496001 | B | US | 2006 |
| FJ496000 | B | US | 2006 |
| FJ495943 | B | US | 2006 |
| FJ495942 | B | US | 2006 |
| FJ495941 | B | US | 2006 |
| FJ495940 | B | US | 2006 |
| FJ495939 | B | US | 2006 |
| FJ495937 | B | US | 2006 |
| FJ495826 | B | US | 2006 |
| FJ495825 | B | US | 2006 |
| FJ495824 | B | US | 2006 |
| FJ495823 | B | US | 2006 |
| FJ495822 | B | US | 2006 |
| FJ495821 | B | US | 2006 |
| FJ495820 | B | US | 2006 |
| FJ495819 | B | US | 2006 |
| FJ495818 | B | US | 2006 |
| FJ469772 | B | US | 2004 |
| FJ469771 | B | US | - |
| FJ469770 | B | US | 2003 |
| FJ469769 | B | US | - |
| FJ469768 | B | US | - |
| FJ469767 | B | US | - |
| FJ469766 | B | US | - |
| FJ469765 | B | US | 2003 |
| FJ469764 | B | US | 2003 |
| FJ469763 | B | US | - |
| FJ469762 | B | US | 2002 |
| FJ469761 | B | US | - |
| FJ469760 | B | US | 2002 |
| FJ469759 | B | US | 2003 |
| FJ469758 | B | US | 2002 |
| FJ469757 | B | US | 2002 |
| FJ469756 | B | US | - |
| FJ469755 | B | US | 2003 |
| FJ469754 | B | US | 2002 |
| FJ469753 | B | US | 2003 |
| FJ469752 | B | US | - |
| FJ469751 | B | US | 2002 |
| FJ469750 | B | US | - |
| FJ469749 | B | US | 2002 |
| FJ469748 | B | US | 2002 |
| FJ469747 | B | US | 2005 |
| FJ469746 | B | US | 2005 |
| FJ469745 | B | US | 2004 |
| FJ469744 | B | US | - |
| FJ469743 | B | US | 2003 |
| FJ469742 | B | US | 2003 |
| FJ469741 | B | US | 2002 |
| FJ469740 | B | US | 2003 |
| FJ469739 | B | US | 2003 |
| FJ469738 | B | US | 2001 |
| FJ469737 | B | US | 2002 |
| FJ469735 | B | US | 2002 |
| FJ469734 | B | US | 2002 |
| FJ469733 | B | US | 2004 |
| FJ469732 | B | US | 2004 |
| FJ469731 | B | US | 2003 |
| FJ469730 | B | US | 2002 |
| FJ469729 | B | US | - |
| FJ469727 | B | US | - |
| FJ469726 | B | US | - |
| FJ469725 | B | US | - |
| FJ469724 | B | US | 2005 |
| FJ469723 | B | US | 2006 |
| FJ469722 | B | US | 2004 |
| FJ469721 | B | US | 2007 |
| FJ469720 | B | US | - |
| FJ469719 | B | US | - |
| FJ469718 | B | US | 2005 |
| FJ469717 | B | US | 2005 |
| FJ469716 | B | US | 2007 |
| FJ469715 | B | US | - |
| FJ469714 | B | US | 2006 |
| FJ469713 | B | US | 2007 |
| FJ469712 | B | US | - |
| FJ469711 | B | US | - |
| FJ469710 | B | US | 2006 |
| FJ469708 | B | US | 2006 |
| FJ469707 | B | US | 2007 |
| FJ469706 | B | US | 2005 |
| FJ469705 | B | US | - |
| FJ469704 | B | US | - |
| FJ469703 | B | US | 2005 |
| FJ469702 | B | US | - |
| FJ469701 | B | US | 2003 |
| FJ469700 | B | US | 2006 |
| FJ469699 | B | US | - |
| FJ469698 | B | US | 2004 |
| FJ469697 | B | US | 2004 |
| FJ469696 | B | US | 2006 |
| FJ469695 | B | US | 2004 |
| FJ469694 | B | US | - |
| FJ469692 | B | US | - |
| FJ469691 | B | US | 2006 |
| FJ469690 | B | US | - |
| FJ469689 | B | US | 2007 |
| FJ469688 | B | US | 2002 |
| FJ469687 | B | US | 2002 |
| FJ469686 | B | US | 2003 |
| FJ469685 | B | US | - |
| FJ469684 | B | US | 2004 |
| FJ469683 | B | US | 2005 |
| FJ469682 | B | US | 2005 |
| FJ460501 | B | HK | 2006 |
| FJ460500 | B | HK | 2006 |
| FJ460499 | B | HK | 2006 |
| FJ388964 | B | CY | 2005 |
| FJ388963 | B | CY | 2005 |
| FJ388962 | B | CY | 2005 |
| FJ388960 | B | CY | 2005 |
| FJ388959 | B | CY | 2005 |
| FJ388958 | B | CY | 2005 |
| FJ388957 | B | CY | 2005 |
| FJ388955 | B | CY | 2006 |
| FJ388947 | B | CY | 2006 |
| FJ388940 | B | CY | 2005 |
| FJ388936 | B | CY | 2005 |
| FJ388935 | B | CY | 2005 |
| FJ388934 | B | CY | 2005 |
| FJ388933 | B | CY | 2005 |
| FJ388931 | B | CY | 2005 |
| FJ388930 | B | CY | 2005 |
| FJ388927 | B | CY | 2005 |
| FJ388924 | B | CY | 2005 |
| FJ388920 | B | CY | 2005 |
| FJ388919 | B | CY | 2005 |
| FJ388916 | B | CY | 2005 |
| FJ388915 | B | CY | 2005 |
| FJ388914 | B | CY | 2005 |
| FJ388912 | B | CY | 2005 |
| FJ388911 | B | CY | 2005 |
| FJ388905 | B | CY | 2005 |
| FJ388904 | B | CY | 2005 |
| FJ388899 | B | CY | 2005 |
| FJ388898 | B | CY | 2005 |
| FJ388895 | B | CY | 2005 |
| FJ388890 | B | CY | 2005 |
| FJ195091 | B | BR | 2005 |
| FJ195090 | B | BR | 2004 |
| FJ195089 | B | BR | 2005 |
| FJ195088 | B | BR | 2005 |
| FJ195086 | B | BR | 2004 |
| FB707281 | B | FR | 1983 |
| EU839610 | B | TT | 2001 |
| EU839609 | B | TT | 2000 |
| EU839608 | B | TT | 2001 |
| EU839607 | B | TT | 2000 |
| EU839606 | B | TT | 2000 |
| EU839605 | B | JM | 2005 |
| EU839604 | B | HT | 2005 |
| EU839603 | B | HT | 2005 |
| EU839602 | B | HT | 2005 |
| EU839601 | B | HT | 2005 |
| EU839600 | B | HT | 2005 |
| EU839598 | B | DO | 2005 |
| EU839597 | B | DO | 2005 |
| EU839596 | B | DO | 2005 |
| EU786680 | B | ES | 2007 |
| EU786679 | B | ES | 2007 |
| EU786678 | B | ES | 2007 |
| EU786675 | B | ES | 2006 |
| EU786674 | B | ES | 2006 |
| EU786672 | B | ES | 2005 |
| EU547186 | B | US | 2004 |
| EF637057 | B | BR | 2003 |
| EF637056 | B | BR | 2003 |
| EF637054 | B | BR | 2003 |
| EF637053 | B | BR | 2003 |
| EF637051 | B | BR | 2003 |
| EF637050 | B | BR | 2003 |
| EF637049 | B | BR | 2003 |
| EF637048 | B | BR | 2003 |
| EF637047 | B | BR | 2003 |
| EF637046 | B | BR | 2003 |
| EF514712 | B | DK | 2001 |
| EF514711 | B | DK | 2001 |
| EF514710 | B | DK | 2001 |
| EF514709 | B | DK | 2001 |
| EF514708 | B | DK | 2001 |
| EF514707 | B | DK | 2001 |
| EF514706 | B | DK | 2001 |
| EF514705 | B | DK | 2001 |
| EF514704 | B | DK | 2001 |
| EF514703 | B | DK | 2004 |
| EF514702 | B | DK | 2004 |
| EF514701 | B | DK | 2004 |
| EF514700 | B | DK | 2004 |
| EF514699 | B | DK | 2004 |
| EF514698 | B | DK | 2004 |
| EF514697 | B | DK | 2004 |
| EF363127 | B | US | 2004 |
| EF363126 | B | US | 2004 |
| EF363125 | B | US | 2004 |
| EF363124 | B | US | 2004 |
| EF363123 | B | US | 2000 |
| EF178358 | B | AU | 2004 |
| EF178354 | B | AU | 2004 |
| EF175209 | B | US | 1998 |
| EF057102 | B | CN | 2001 |
| DQ990880 | B | CN | 2005 |
| DQ886037 | B | US | 2005 |
| DQ854716 | B | ES | 1989 |
| DQ854715 | B | ES | 1989 |
| DQ854714 | B | ES | 1989 |
| DQ853463 | B | US | 1998 |
| DQ853461 | B | US | 1998 |
| DQ853460 | B | US | 1998 |
| DQ853459 | B | US | 1998 |
| DQ853455 | B | US | 1998 |
| DQ853454 | B | US | 2000 |
| DQ853453 | B | US | 2000 |
| DQ853452 | B | US | 2000 |
| DQ853451 | B | US | 2000 |
| DQ853450 | B | US | 2000 |
| DQ853449 | B | US | 2000 |
| DQ853448 | B | US | 2002 |
| DQ853447 | B | US | 2002 |
| DQ853446 | B | US | 2002 |
| DQ853445 | B | US | 2002 |
| DQ853444 | B | US | 2002 |
| DQ853443 | B | US | 2002 |
| DQ853442 | B | US | 2002 |
| DQ853441 | B | US | 2002 |
| DQ853440 | B | US | 2002 |
| DQ853439 | B | US | 2002 |
| DQ853438 | B | US | 2000 |
| DQ853437 | B | US | 2000 |
| DQ853436 | B | US | 2000 |
| DQ837381 | B | KR | 2005 |
| DQ823364 | B | UA | 2001 |
| DQ823363 | B | UA | 2001 |
| DQ823362 | B | UA | 2001 |
| DQ676886 | B | AU | 2003 |
| DQ676883 | B | AU | 2002 |
| DQ676880 | B | AU | 2003 |
| DQ676878 | B | AU | 2003 |
| DQ676874 | B | AU | 2002 |
| DQ676871 | B | AU | 2004 |
| DQ672623 | B | IT | 2005 |
| DQ487191 | B | US | 1996 |
| DQ487190 | B | US | 1993 |
| DQ487189 | B | US | 1995 |
| DQ487188 | B | US | 1993 |
| DQ396398 | B | ZA | 2003 |
| DQ383752 | B | AR | 2004 |
| DQ383751 | B | AR | 2004 |
| DQ383750 | B | AR | 2004 |
| DQ383749 | B | AR | 2003 |
| DQ383748 | B | AR | 2003 |
| DQ383746 | B | AR | 2002 |
| DQ358810 | B | BR | 2002 |
| DQ358809 | B | BR | 2002 |
| DQ358808 | B | BR | 2002 |
| DQ358805 | B | BR | 2002 |
| DQ354119 | B | TH | 1996 |
| DQ354118 | B | TH | 1996 |
| DQ354116 | B | TH | 1996 |
| DQ354114 | B | TH | 1996 |
| DQ354112 | B | TH | 1996 |
| DQ295196 | B | KR | 2004 |
| DQ295195 | B | KR | 2004 |
| DQ295194 | B | KR | 2004 |
| DQ295193 | B | KR | 2004 |
| DQ295192 | B | KR | 2004 |
| DQ207943 | B | GE | 1998 |
| DQ207942 | B | GE | 2003 |
| DQ207940 | B | GE | 2003 |
| DQ007903 | B | CN | 2002 |
| DQ007902 | B | CN | 2002 |
| DQ007901 | B | CN | 2002 |
| D86069 | B | FR | 1983 |
| D86068 | B | FR | 1983 |
| D10112 | B | GB | 1983 |
| BD298114 | B | FR | 1983 |
| BD238372 | B | FR | 1985 |
| AY970950 | B | NL | 1996 |
| AY970949 | B | NL | 1996 |
| AY970948 | B | NL | 1996 |
| AY970947 | B | NL | 1986 |
| AY970946 | B | NL | 1986 |
| AY945711 | B | TH | 1999 |
| AY945710 | B | TH | 2000 |
| AY839827 | B | KR | 2004 |
| AY835781 | B | US | 1983 |
| AY835780 | B | US | 1986 |
| AY835779 | B | US | 1984 |
| AY835778 | B | US | 1986 |
| AY835777 | B | US | 1983 |
| AY835776 | B | US | 1993 |
| AY835775 | B | US | 1986 |
| AY835774 | B | US | 1986 |
| AY835773 | B | US | 1994 |
| AY835772 | B | US | 1997 |
| AY835771 | B | US | 1986 |
| AY835770 | B | US | 1983 |
| AY835769 | B | US | 1985 |
| AY835768 | B | US | 1995 |
| AY835767 | B | US | 1993 |
| AY835766 | B | US | 1989 |
| AY835765 | B | US | 1984 |
| AY835763 | B | US | 1988 |
| AY835762 | B | US | 1984 |
| AY835761 | B | US | 1991 |
| AY835760 | B | US | 1987 |
| AY835759 | B | US | 1982 |
| AY835758 | B | US | 1987 |
| AY835756 | B | US | 1986 |
| AY835755 | B | US | 1985 |
| AY835754 | B | US | 1983 |
| AY835753 | B | US | 1996 |
| AY835752 | B | US | 1992 |
| AY835750 | B | US | 1989 |
| AY835749 | B | US | 1986 |
| AY835748 | B | US | 1983 |
| AY819715 | B | RU | 2004 |
| AY818644 | B | AU | 2004 |
| AY795905 | B | YE | 2002 |
| AY795904 | B | YE | 2002 |
| AY781127 | B | UY | 2001 |
| AY781126 | B | UY | 2001 |
| AY779564 | B | CA | 1996 |
| AY779563 | B | CA | 1996 |
| AY779562 | B | CA | 1993 |
| AY779561 | B | CA | 1992 |
| AY779560 | B | CA | 1994 |
| AY779559 | B | CA | 1992 |
| AY779558 | B | CA | 1999 |
| AY779557 | B | CA | 1998 |
| AY779555 | B | CA | 1998 |
| AY779554 | B | CA | 1998 |
| AY779553 | B | CA | 1997 |
| AY779552 | B | CA | 2000 |
| AY779551 | B | CA | 1999 |
| AY779550 | B | CA | 1998 |
| AY751407 | B | RU | 2004 |
| AY751406 | B | RU | 2004 |
| AY713412 | B | US | 1990 |
| AY713411 | B | FR | 1992 |
| AY713410 | B | US | 1994 |
| AY713409 | B | US | 1985 |
| AY713408 | B | TH | 1996 |
| AY682547 | B | RU | 2004 |
| AY586543 | B | CU | 1999 |
| AY586542 | B | CU | 1999 |
| AY561240 | B | CO | 2001 |
| AY561238 | B | CO | 2001 |
| AY561237 | B | CO | 2001 |
| AY561236 | B | CO | 2001 |
| AY560110 | B | US | 1998 |
| AY560109 | B | US | 1998 |
| AY560108 | B | US | 1998 |
| AY560107 | B | US | 1998 |
| AY423387 | B | NL | 2000 |
| AY423386 | B | NL | 2000 |
| AY423385 | B | NL | 2000 |
| AY423384 | B | NL | 2000 |
| AY423383 | B | NL | 2000 |
| AY423382 | B | NL | 2000 |
| AY423381 | B | NL | 1999 |
| AY352275 | B | US | 1984 |
| AY332237 | B | US | 1996 |
| AY332236 | B | US | 1997 |
| AY331297 | B | US | 1999 |
| AY331296 | B | US | 1999 |
| AY331295 | B | US | 1998 |
| AY331294 | B | US | 1998 |
| AY331293 | B | US | 1996 |
| AY331292 | B | US | 1996 |
| AY331291 | B | US | 1996 |
| AY331290 | B | US | 1997 |
| AY331289 | B | US | 1997 |
| AY331288 | B | US | 1997 |
| AY331287 | B | US | 1997 |
| AY331286 | B | US | 1997 |
| AY331285 | B | US | 1997 |
| AY331284 | B | US | 1997 |
| AY331283 | B | US | 1997 |
| AY331282 | B | US | 1997 |
| AY314063 | B | CA | 1996 |
| AY314044 | B | CA | 1996 |
| AY308762 | B | US | 1996 |
| AY308761 | B | US | 1996 |
| AY308760 | B | US | 1996 |
| AY180905 | B | CN | 2001 |
| AY173956 | B | BR | 1989 |
| AY173955 | B | US | 1990 |
| AY173954 | B | US | 1990 |
| AY173952 | B | US | 1990 |
| AY173951 | B | TH | 1990 |
| AY037282 | B | AR | 1999 |
| AY037270 | B | BO | 1999 |
| AY037269 | B | AR | 2000 |
| AY037268 | B | AR | 1998 |
| AX078307 | B | FR | 1983 |
| AX032749 | B | FR | 1985 |
| AJ271445 | B | GB | 1986 |
| AJ006287 | B | ES | 1989 |
| AF538307 | B | AU | 1995 |
| AF538306 | B | AU | 1995 |
| AF538305 | B | AU | 1995 |
| AF538304 | B | AU | 1995 |
| AF538303 | B | AU | 1999 |
| AF538302 | B | AU | 1999 |
| AF286365 | B | US | 1988 |
| AF256204 | B | ES | 1989 |
| AF224507 | B | KR | 1997 |
| AF086817 | B | TW | 1994 |
| AF070521 | B | FR | 1985 |
| AF069140 | B | US | 1991 |
| AF042106 | B | AU | 1993 |
| AF042105 | B | AU | 1996 |
| AF042104 | B | AU | 1996 |
| AF042103 | B | AU | 1995 |
| AF042102 | B | AU | 1993 |
| AF042101 | B | AU | 1987 |
| AF042100 | B | AU | 1986 |
| AF004394 | B | US | 1986 |
| AF003887 | B | FR | 1985 |
| AB731670 | B | JP | 2011 |
| AB731668 | B | JP | 2009 |
| AB731667 | B | JP | 2009 |
| AB731664 | B | JP | 2008 |
| AB731663 | B | JP | 2008 |
| AB485642 | B | BR | 1990 |
| AB485641 | B | BR | 1990 |
| AB485639 | B | US | 1991 |
| AB485638 | B | US | 1991 |
| AB480698 | B | JP | 1998 |
| AB480697 | B | JP | 2004 |
| AB480695 | B | JP | 2004 |
| AB480694 | B | JP | 2004 |
| AB480693 | B | JP | 2004 |
| AB480692 | B | JP | 2004 |
| AB428562 | B | JP | 2006 |
| AB428561 | B | JP | 2006 |
| AB428560 | B | JP | 2005 |
| AB428559 | B | JP | 2004 |
| AB428558 | B | JP | 2003 |
| AB428557 | B | JP | 2005 |
| AB428556 | B | JP | 2005 |
| AB428555 | B | JP | 2003 |
| AB428554 | B | JP | 2002 |
| AB428553 | B | JP | 2002 |
| AB428552 | B | JP | 2001 |
| AB428551 | B | JP | 2000 |
| AB289590 | B | JP | 2001 |
| AB289588 | B | JP | 2000 |
| AB289587 | B | JP | 2000 |
| AB287372 | B | JP | 2000 |
| AB287370 | B | JP | 1999 |
| AB287368 | B | JP | 2005 |
| AB287366 | B | JP | 2005 |
| AB287364 | B | JP | 2005 |
| AB287363 | B | JP | 2005 |
| AB286955 | B | JP | 2004 |
| AB253432 | B | US | 1985 |
| AB221125 | B | JP | 2004 |
| AB097870 | B | MM | 1999 |
| AB078005 | B | US | 1997 |
| A07867 | B | FR | 1983 |
| A04321 | B | FR | 1983 |

**Subtype C**

| Accession number | Clade | Country | years |
| --- | --- | --- | --- |
| EU786681 | C | ES | 2008 |
| AY713417 | C | ET | 2002 |
| AB485643 | C | DJ | - |
| AB485644 | C | DJ | - |
| AY255823 | C | IL | 1999 |
| KF526218 | C | US | 2011 |
| U46016 | C | ET | 1986 |
| AF286233 | C | IL | 1998 |
| AY255825 | C | IL | 1999 |
| AY255826 | C | IL | 1999 |
| FJ388952 | C | CY | 2006 |
| JF683776 | C | CY | 2008 |
| JF683777 | C | CY | 2008 |
| KC156217 | C | MW | 2008 |
| AY901969 | C | ZA | 2003 |
| JX140663 | C | BR | 2007 |
| AY563170 | C | AR | 2001 |
| AF286228 | C | BR | 1998 |
| U52953 | C | BR | 1992 |
| AY563169 | C | UY | 2001 |
| AY727525 | C | BR | 2004 |
| AY727524 | C | BR | 2004 |
| AY727523 | C | BR | 2004 |
| AY727522 | C | BR | 2004 |
| AY945738 | C | KE | 1991 |
| GQ999983 | C | ZA | 2005 |
| FJ496185 | C | ZM | 2003 |
| AY703910 | C | ZA | 2004 |
| FJ388901 | C | CY | 2005 |
| KC870038 | C | CN | 2010 |
| KC156210 | C | IN | 2009 |
| AB023804 | C | IN | 1993 |
| AY713414 | C | IN | 1994 |
| AY967806 | C | CN | 1998 |
| AB097871 | C | MM | 1999 |
| AF067155 | C | IN | 1995 |
| AF067159 | C | IN | 1994 |
| AF067154 | C | IN | 1993 |
| KC898980 | C | CN | 2006 |
| KF835522 | C | CN | 2007 |
| KF250404 | C | CN | 2009 |
| KC898995 | C | CN | 2009 |
| KC898996 | C | CN | 2009 |
| KF250403 | C | CN | 2009 |
| AF286232 | C | IN | 1998 |
| AF286231 | C | IN | 1998 |
| EF694032 | C | IN | - |
| EF694033 | C | IN | - |
| KF835515 | C | CN | 2007 |
| AY049709 | C | IN | 1999 |
| KF766539 | C | IN | 2004 |
| KF766540 | C | IN | 2005 |
| KF766541 | C | IN | 2005 |
| AF110968 | C | BW | 1996 |
| AF443095 | C | BW | 2000 |
| AF110976 | C | BW | 1996 |
| AF110978 | C | BW | 1996 |
| AF110977 | C | BW | 1996 |
| AF457054 | C | KE | 2000 |
| AB485645 | C | ZM | 1989 |
| AY713415 | C | SO | 1989 |
| AY228556 | C | ZA | 1999 |
| AY228557 | C | ZA | 2001 |
| AY253310 | C | TZ | 2001 |
| AF286224 | C | ZM | 1996 |
| AY734550 | C | TZ | 2002 |
| AY043173 | C | ZA | 1998 |
| AY043175 | C | ZA | 1999 |
| AY463234 | C | ZA | 2000 |
| AY463227 | C | ZA | 2000 |
| DQ275645 | C | ZA | 2003 |
| KC156125 | C | ZA | 2007 |
| AY838565 | C | ZA | 2000 |
| AY878055 | C | ZA | 2004 |
| DQ056406 | C | ZA | 2004 |
| GQ999974 | C | ZA | 2005 |
| DQ351216 | C | ZA | 2003 |
| DQ093588 | C | ZA | 2004 |
| AY463222 | C | ZA | 2000 |
| DQ275646 | C | ZA | 2003 |
| DQ396378 | C | ZA | 2003 |
| AY878059 | C | ZA | 2004 |
| DQ396368 | C | ZA | 2003 |
| DQ011170 | C | ZA | 2004 |
| AY772693 | C | ZA | 2003 |
| DQ164110 | C | ZA | 2004 |
| AY463224 | C | ZA | 2000 |
| EU293447 | C | ZA | 1999 |
| AY463226 | C | ZA | 2000 |
| AY463231 | C | ZA | 2000 |
| AY772696 | C | ZA | 2003 |
| DQ351218 | C | ZA | 2002 |
| DQ396394 | C | ZA | 2004 |
| DQ275642 | C | ZA | 2003 |
| DQ275643 | C | ZA | 2003 |
| DQ351219 | C | ZA | 2003 |
| DQ396371 | C | ZA | 2003 |
| AY772690 | C | ZA | 2003 |
| DQ396376 | C | ZA | 2003 |
| AY901968 | C | ZA | 2003 |
| DQ164116 | C | ZA | 2004 |
| DQ275651 | C | ZA | 2003 |
| DQ351221 | C | ZA | 2004 |
| DQ164114 | C | ZA | 2004 |
| AY878071 | C | ZA | 2004 |
| DQ164104 | C | ZA | 2003 |
| DQ164111 | C | ZA | 2004 |
| AF286227 | C | ZA | 1997 |
| AY463219 | C | ZA | 2000 |
| AY901977 | C | ZA | 2004 |
| DQ396370 | C | ZA | 2003 |
| GQ999980 | C | ZA | 2005 |
| AY463218 | C | ZA | 2000 |
| AY463228 | C | ZA | 2000 |
| DQ164113 | C | ZA | 2003 |
| DQ011172 | C | ZA | 2004 |
| AY463221 | C | ZA | 2000 |
| AY878072 | C | ZA | 2004 |
| AY463217 | C | ZA | 2000 |
| GQ999984 | C | ZA | 2005 |
| DQ011174 | C | ZA | 2004 |
| DQ093587 | C | ZA | 2004 |
| DQ369977 | C | ZA | 2003 |
| DQ396383 | C | ZA | 2004 |
| DQ351222 | C | ZA | 2002 |
| DQ351223 | C | ZA | 2003 |
| AY463220 | C | ZA | 2000 |
| AY463223 | C | ZA | 2001 |
| DQ093591 | C | ZA | 2003 |
| DQ369983 | C | ZA | 2005 |
| DQ369984 | C | ZA | 2003 |
| DQ396369 | C | ZA | 2003 |
| AY878058 | C | ZA | 2004 |
| DQ369979 | C | ZA | 2003 |
| DQ056409 | C | ZA | 2004 |
| DQ351227 | C | ZA | 2003 |
| DQ093593 | C | ZA | 2003 |
| DQ275654 | C | ZA | 2003 |
| DQ164112 | C | ZA | 2004 |
| AY463237 | C | ZA | 2001 |
| DQ093596 | C | ZA | 2003 |
| DQ978981 | C | ZA | 2003 |
| AY901978 | C | ZA | 2004 |
| DQ164109 | C | ZA | 2004 |
| DQ396375 | C | ZA | 2003 |
| DQ369978 | C | ZA | 2003 |
| DQ351220 | C | ZA | 2002 |
| DQ369976 | C | ZA | 2003 |
| AY043174 | C | ZA | 1999 |
| DQ351228 | C | ZA | 2003 |
| AY118165 | C | ZA | 1997 |
| AY158534 | C | ZA | 1998 |
| DQ056415 | C | ZA | 2004 |
| DQ396388 | C | ZA | 2003 |
| AF110959 | C | BW | 1996 |
| AF110960 | C | BW | 1996 |
| AF110961 | C | BW | 1996 |
| AY158535 | C | ZA | 1998 |
| AF443084 | C | BW | 1999 |
| AF443099 | C | BW | 2000 |
| DQ164129 | C | ZA | 2004 |
| AY901973 | C | ZA | 2004 |
| DQ093590 | C | ZA | 2004 |
| AY878065 | C | ZA | 2003 |
| DQ445631 | C | ZA | 2004 |
| DQ396364 | C | ZA | 2003 |
| AY901970 | C | ZA | 2003 |
| AY901979 | C | ZA | 2004 |
| DQ396385 | C | ZA | 2003 |
| DQ369989 | C | ZA | 2003 |
| AY901971 | C | ZA | 2003 |
| DQ164106 | C | ZA | 2003 |
| AF110973 | C | BW | 1996 |
| AF110974 | C | BW | 1996 |
| AF110975 | C | BW | 1996 |
| AF443102 | C | BW | 2000 |
| DQ093604 | C | ZA | 2004 |
| AF443109 | C | BW | 2000 |
| DQ396384 | C | ZA | 2003 |
| DQ369987 | C | ZA | 2003 |
| AF443085 | C | BW | 1999 |
| AF443113 | C | BW | 2000 |
| DQ396391 | C | ZA | 2003 |
| DQ011173 | C | ZA | 2004 |
| GQ999985 | C | ZA | 2005 |
| GQ999981 | C | ZA | 2005 |
| DQ093601 | C | ZA | 2003 |
| DQ275658 | C | ZA | 2003 |
| DQ369990 | C | ZA | 2003 |
| DQ396372 | C | ZA | 2005 |
| AY878056 | C | ZA | 2003 |
| DQ396374 | C | ZA | 2003 |
| AF286223 | C | IN | 1994 |
| AY444801 | C | US | 1998 |
| EF514713 | C | DK | 2001 |
| AF286234 | C | TZ | 1998 |
| AF443089 | C | BW | 2000 |
| AY463230 | C | ZA | 2000 |
| DQ351225 | C | ZA | 2003 |
| DQ275659 | C | ZA | 2004 |
| AY878054 | C | ZA | 2004 |
| AY901965 | C | ZA | 2003 |
| DQ396395 | C | ZA | 2003 |
| AY901976 | C | ZA | 2004 |
| JX140668 | C | ZA | 2009 |
| DQ396367 | C | ZA | 2003 |
| DQ275653 | C | ZA | 2003 |
| EU293448 | C | ZA | 1999 |
| DQ369981 | C | ZA | 2003 |
| DQ056412 | C | ZA | 2004 |
| GQ999978 | C | ZA | 2005 |
| DQ011176 | C | ZA | 2003 |
| DQ275657 | C | ZA | 2003 |
| DQ369988 | C | ZA | 2003 |
| GQ999990 | C | ZA | 2005 |
| DQ093592 | C | ZA | 2003 |
| KC156130 | C | ZA | 2007 |
| AY772699 | C | ZA | 2004 |
| AY878057 | C | ZA | 2003 |
| AY901975 | C | ZA | 2003 |
| AY585266 | C | ZA | 2000 |
| DQ351226 | C | ZA | 2003 |
| AY901967 | C | ZA | 2003 |
| DQ445633 | C | ZA | 2003 |
| AY901981 | C | ZA | 2003 |
| AY772692 | C | ZA | 2003 |
| GQ999982 | C | ZA | 2005 |
| DQ369992 | C | ZA | 2005 |
| AY772695 | C | ZA | 2003 |
| DQ351233 | C | ZA | 2003 |
| AY878063 | C | ZA | 2003 |
| DQ396382 | C | ZA | 2004 |
| DQ164115 | C | ZA | 2004 |
| DQ093600 | C | ZA | 2004 |
| AF411966 | C | ZA | 1999 |
| DQ275661 | C | ZA | 2003 |
| DQ056408 | C | ZA | 2003 |
| DQ164105 | C | ZA | 2004 |
| DQ093599 | C | ZA | 2004 |
| DQ011166 | C | ZA | 2004 |
| AY703908 | C | ZA | 2003 |
| DQ011169 | C | ZA | 2003 |
| DQ396396 | C | ZA | 2004 |
| GQ999987 | C | ZA | 2005 |
| AY463225 | C | ZA | 2000 |
| DQ164107 | C | ZA | 2004 |
| AY901980 | C | ZA | 2004 |
| DQ396373 | C | ZA | 2003 |
| GQ999973 | C | ZA | 2004 |
| DQ164108 | C | ZA | 2003 |
| DQ275644 | C | ZA | 2003 |
| DQ396377 | C | ZA | 2003 |
| DQ275652 | C | ZA | 2003 |
| AY878068 | C | ZA | 2003 |
| DQ093594 | C | ZA | 2004 |
| DQ369982 | C | ZA | 2005 |
| AY463229 | C | ZA | 2000 |
| DQ093597 | C | ZA | 2003 |
| AY585265 | C | ZA | 2000 |
| AY838568 | C | ZA | 2000 |
| DQ164117 | C | ZA | 2004 |
| DQ275647 | C | ZA | 2002 |
| DQ275648 | C | ZA | 2002 |
| DQ351231 | C | ZA | 2003 |
| DQ396390 | C | ZA | 2003 |
| AY901966 | C | ZA | 2003 |
| DQ164122 | C | ZA | 2004 |
| DQ445635 | C | ZA | 2003 |
| DQ396381 | C | ZA | 2004 |
| DQ093586 | C | ZA | 2004 |
| DQ011175 | C | ZA | 2003 |
| AF443082 | C | BW | 1998 |
| DQ164118 | C | ZA | 2004 |
| AY118166 | C | ZA | 1997 |
| DQ056413 | C | ZA | 2004 |
| EU293445 | C | ZA | 1999 |
| DQ396397 | C | ZA | 2004 |
| DQ275656 | C | ZA | 2003 |
| DQ369997 | C | ZA | 2004 |
| DQ369986 | C | ZA | 2003 |
| DQ011165 | C | ZA | 2003 |
| DQ056418 | C | ZA | 2004 |
| AY772694 | C | ZA | 2003 |
| GQ999977 | C | ZA | 2005 |
| DQ011179 | C | ZA | 2004 |
| DQ445637 | C | ZA | 2004 |
| GQ999975 | C | ZA | 2004 |
| AY795906 | C | YE | 2002 |
| AY255824 | C | IL | 1999 |
| AY713416 | C | SN | 1990 |
| AY463233 | C | ZA | 2000 |
| AF110969 | C | BW | 1996 |
| AF110970 | C | BW | 1996 |
| AF110971 | C | BW | 1996 |
| AF443074 | C | BW | 1996 |
| AF443077 | C | BW | 1998 |
| AF443103 | C | BW | 2000 |
| JN188292 | C | ZA | 1990 |
| AF443088 | C | BW | 2000 |
| AF443097 | C | BW | 2000 |
| AF443100 | C | BW | 2000 |
| FJ496195 | C | ZM | 2003 |
| AY162225 | C | ZA | 1998 |
| AY703911 | C | ZA | 2004 |
| DQ011171 | C | ZA | 2004 |
| AB254148 | C | ZM | 2002 |
| AF443094 | C | BW | 2000 |
| AF443096 | C | BW | 2000 |
| DQ056416 | C | ZA | 2004 |
| AF443086 | C | BW | 1999 |
| AB254141 | C | ZM | 2002 |
| AB254146 | C | ZM | 2002 |
| AB254147 | C | ZM | 2002 |
| AF286225 | C | ZM | 1996 |
| DQ164127 | C | ZA | 2004 |
| DQ351217 | C | ZA | 2004 |
| AY253308 | C | TZ | 2001 |
| AF286235 | C | TZ | 1998 |
| AF361874 | C | TZ | 1997 |
| AY253320 | C | TZ | 2001 |
| AF443079 | C | BW | 1998 |
| AF443104 | C | BW | 2000 |
| DQ369996 | C | ZA | 2003 |
| AF110962 | C | BW | 1996 |
| AF110964 | C | BW | 1996 |
| AF110966 | C | BW | 1996 |
| AF110965 | C | BW | 1996 |
| AY805330 | C | ZM | - |
| EU293444 | C | ZA | 1999 |
| DQ093589 | C | ZA | 2003 |
| AY158533 | C | ZA | 1998 |
| AY838567 | C | ZA | 2000 |
| DQ275649 | C | ZA | 2003 |
| DQ396366 | C | ZA | 2004 |
| DQ011177 | C | ZA | 2004 |
| DQ275650 | C | ZA | 2003 |
| AY713413 | C | MW | 1993 |
| DQ351237 | C | ZA | 2003 |
| DQ396380 | C | ZA | 2003 |
| AY253313 | C | TZ | 2001 |
| AY253304 | C | TZ | 2001 |
| AY253312 | C | TZ | 2001 |
| AF443080 | C | BW | 1998 |
| AY162224 | C | ZA | 1998 |
| KC156212 | C | MW | 2007 |
| AY463236 | C | ZA | 2000 |
| AY772700 | C | ZA | 2003 |
| AF411967 | C | ZA | 1999 |
| AY162223 | C | ZA | 1998 |
| AY253321 | C | TZ | 2001 |
| AF110972 | C | BW | 1996 |
| AY463232 | C | ZA | 2000 |
| DQ396365 | C | ZA | 2003 |
| DQ093595 | C | ZA | 2004 |
| DQ275664 | C | ZA | 2003 |
| DQ369985 | C | ZA | 2003 |
| DQ369991 | C | ZA | 2005 |
| GQ999991 | C | ZA | 2005 |
| AF361875 | C | TZ | 1997 |
| AF443112 | C | BW | 2000 |
| AY444800 | C | US | 1998 |
| AF443081 | C | BW | 1998 |
| DQ369993 | C | ZA | 2004 |
| AY253322 | C | TZ | 2001 |
| DQ207941 | C | GE | 2003 |
| AY734556 | C | TZ | 2002 |
| AY043176 | C | ZA | 1998 |
| EU293446 | C | ZA | 1999 |
| DQ351229 | C | ZA | 2003 |
| AY878060 | C | ZA | 2003 |
| AF290027 | C | BW | 1996 |
| AF290028 | C | BW | 1996 |
| AF290029 | C | BW | 1996 |
| AF443093 | C | BW | 2000 |
| AF443110 | C | BW | 2000 |
| GQ999972 | C | ZA | 2005 |
| DQ011167 | C | ZA | 2004 |
| DQ351234 | C | ZA | 2003 |
| JX974246 | C | ZA | 2007 |
| KC156126 | C | ZA | 2007 |
| KC156115 | C | ZA | 2007 |
| AY878062 | C | ZA | 2004 |
| AF443078 | C | BW | 1998 |
| DQ056404 | C | ZA | 2003 |
| AY703909 | C | ZA | 2004 |
| DQ056405 | C | ZA | 2004 |
| DQ396387 | C | ZA | 2004 |
| AF443101 | C | BW | 2000 |
| DQ396386 | C | ZA | 2003 |
| DQ011180 | C | ZA | 2004 |
| EU884500 | C | ES | 2007 |
| JX140662 | C | ES | 2007 |
| KF527172 | C | MW | 2008 |
| KF527173 | C | MW | 2008 |
| DQ445634 | C | ZA | 2003 |
| DQ396393 | C | ZA | 2004 |
| KC156127 | C | ZA | 2007 |
| KC156128 | C | ZA | 2008 |
| AF443107 | C | BW | 2000 |
| AF443076 | C | BW | 1998 |
| AF443090 | C | BW | 2000 |
| DQ445632 | C | ZA | 2003 |
| EU293450 | C | ZA | 1999 |
| KC156215 | C | MW | 2008 |
| AF443098 | C | BW | 2000 |
| DQ093598 | C | ZA | 2004 |
| DQ369994 | C | ZA | 2005 |
| KC156114 | C | MW | 2007 |
| AY878061 | C | ZA | 2003 |
| AY901974 | C | ZA | 2004 |
| DQ396389 | C | ZA | 2003 |
| DQ164119 | C | ZA | 2004 |
| DQ275660 | C | ZA | 2003 |
| DQ396392 | C | ZA | 2003 |
| DQ011178 | C | ZA | 2004 |
| DQ275655 | C | ZA | 2003 |
| AF443111 | C | BW | 2000 |
| EU293449 | C | ZA | 1999 |
| AY838566 | C | ZA | 2000 |
| AF443083 | C | BW | 1999 |
| AF443105 | C | BW | 2000 |
| AF443108 | C | BW | 2000 |
| KF716466 | C | ZM | 2009 |
| AY253317 | C | TZ | 2001 |
| KC156219 | C | MW | 2008 |
| DQ056411 | C | ZA | 2003 |
| AY878070 | C | ZA | 2003 |
| DQ351230 | C | ZA | 2003 |
| JX140666 | C | ZA | 2008 |
| AY878064 | C | ZA | 2003 |
| DQ396399 | C | ZA | 2003 |
| KC156116 | C | ZA | 2008 |
| KC156129 | C | ZA | 2007 |
| GQ999989 | C | ZA | 2005 |
| DQ351224 | C | ZA | 2003 |
| GQ999976 | C | ZA | 2005 |
| KC156120 | C | MW | 2007 |
| KC156119 | C | MW | 2007 |
| DQ093607 | C | ZA | 2003 |
| KC156221 | C | ZA | 2008 |
| AF443092 | C | BW | 2000 |
| KF716467 | C | ZM | 2011 |
| AY253303 | C | TZ | 2001 |
| AB254149 | C | ZM | 2002 |
| EU786673 | C | ES | 2006 |
| FJ670521 | C | ES | 2005 |
| AY734558 | C | TZ | 2002 |
| AB254155 | C | ZM | 2002 |
| AY734560 | C | TZ | 2002 |
| AF443087 | C | BW | 1999 |
| AF110980 | C | BW | 1996 |
| AF443091 | C | BW | 2000 |
| AY772691 | C | ZA | 2003 |
| DQ351235 | C | ZA | 2002 |
| DQ093602 | C | ZA | 2004 |
| DQ093605 | C | ZA | 2004 |
| AF443075 | C | BW | 1996 |
| FJ388913 | C | CY | 2005 |
| JX140669 | C | ZA | 2010 |
| GQ999979 | C | ZA | 2005 |
| AY734551 | C | TZ | 2002 |
| KC156214 | C | MW | 2009 |
| KC156122 | C | MW | 2007 |
| KC156213 | C | MW | 2007 |
| GQ999986 | C | ZA | 2005 |
| AF443114 | C | BW | 2000 |
| DQ164126 | C | ZA | 2004 |
| DQ369995 | C | ZA | 2002 |
| DQ056410 | C | ZA | 2003 |
| AY901972 | C | ZA | 2003 |
| JX140667 | C | ZA | 2009 |
| DQ351232 | C | ZA | 2004 |
| KC156220 | C | TZ | 2008 |
| DQ164121 | C | ZA | 2004 |
| KC156211 | C | MW | 2007 |
| DQ369980 | C | ZA | 2003 |
| AF443115 | C | BW | 2000 |
| FJ496209 | C | ZM | 2003 |
| DQ093585 | C | ZA | 2004 |
| DQ056417 | C | ZA | 2004 |
| EF469243 | C | IN | 2003 |
| KC156218 | C | MW | 2008 |
| AB254142 | C | ZM | 2002 |
| AY734559 | C | TZ | 2002 |
| AY772698 | C | ZA | 2004 |
| JF683770 | C | CY | 2008 |
| AY253307 | C | TZ | 2001 |
| JF683755 | C | CY | 2007 |

**Subtype D**

| Accession number | Clade | Country | years |
| --- | --- | --- | --- |
| AY773339 | D2 | ZA | 1985 |
| AY773340 | D2 | ZA | 1985 |
| DQ054367 | D1 | KR | 2004 |
| AY773341 | D2 | ZA | 1986 |
| JX140670 | D3 | CM | 2010 |
| FJ388945 | D3 | CY | 2006 |
| KJ787684 | D2 | BR | 2010 |
| AY322189 | D1 | KE | 1997 |
| KF716476 | D1 | KE | 2011 |
| KF716480 | D1 | UG | 2011 |
| JX236672 | D1 | UG | 2008 |
| JQ403079 | D1 | US | 2007 |
| KF716479 | D1 | UG | 2010 |
| KF716502 | D1 | UG | 2007 |
| JX236679 | D1 | UG | 2007 |
| AF484519 | D1 | UG | 1999 |
| AF484486 | D | UG | 1999 |
| AF484505 | D1 | UG | 1998 |
| AF484485 | D1 | UG | 1999 |
| AF484498 | D1 | UG | 1999 |
| AF484518 | D1 | UG | 1999 |
| AF484495 | D1 | UG | 1999 |
| AF484502 | D1 | UG | 1998 |
| AF484511 | D1 | UG | 1998 |
| AF484490 | D1 | UG | 1999 |
| AY304496 | D1 | UG | 1999 |
| AF484487 | D1 | UG | 1999 |
| AY795903 | D1 | YE | 2001 |
| AF484477 | D1 | UG | 1999 |
| AF484499 | D1 | UG | 1999 |
| AF457090 | D1 | KE | 2001 |
| JX236668 | D1 | UG | 2005 |
| AF484513 | D1 | UG | 1998 |
| JX236670 | D1 | UG | 2007 |
| AF484489 | D1 | UG | 1999 |
| AF484506 | D1 | UG | 1998 |
| AY713418 | D1 | UG | 1993 |
| U88824 | D1 | UG | 1994 |
| AF484483 | D1 | UG | 1999 |
| AF484515 | D1 | UG | 1999 |
| AF484497 | D1 | UG | 1999 |
| AF484516 | D1 | UG | 1998 |
| AF484494 | D1 | UG | 1999 |
| AF484481 | D1 | UG | 1999 |
| AF484480 | D1 | UG | 1999 |
| AB485650 | D1 | UG | 1991 |
| JX236673 | D1 | UG | 2007 |
| AF484514 | D1 | UG | 1998 |
| AJ320484 | D1 | UG | 1992 |
| AF484504 | D1 | UG | 1998 |
| AY253311 | D1 | TZ | 2001 |
| AB485648 | D1 | SN | 1990 |
| KJ787683 | D2 | BR | 2010 |
| U88822 | D3 | CD | 1984 |
| A34828 | D2 | CD | 1983 |
| M22639 | D2 | CD | 1985 |
| A07108 | D2 | CD | 1983 |
| AY773338 | D2 | ZA | 1984 |
| EF633445 | D2 | ZA | 1990 |
| AY371157 | D3 | CM | 2001 |
| AY371156 | D3 | CM | 2001 |
| AY371155 | D3 | CM | 2001 |
| AJ519489 | D3 | TD | 1999 |
| AJ488927 | D3 | TD | 1999 |
| AJ488926 | D3 | TD | 1999 |
| AY795907 | D3 | YE | 2002 |

**Subtype F**

| Accession number | Clade | Country | years |
| --- | --- | --- | --- |
| AJ249236 | F | CM | 1995 |
| AJ249237 | F | CM | 1995 |
| AF377956 | F | CM | 1997 |
| AY371158 | F | CM | 2002 |
| JX140673 | F | CM | 2010 |
| JX140672 | F | CM | 2010 |
| DQ189088 | F | AR | 2002 |
| AY173958 | F | BR | 1989 |
| AY173957 | F | BR | 1989 |
| FJ771006 | F | BR | 2002 |
| AF005494 | F | BR | 1993 |
| AF077336 | F | BE | 1993 |
| AF075703 | F | FI | 1993 |
| FJ771010 | F | BR | 2007 |
| JX140671 | F | ES | 2011 |
| JF683771 | F | CY | 2008 |
| AJ249238 | F | FR | 1996 |
| AB485658 | F | RO | 1996 |
| DQ979023 | F | ES | - |
| DQ979024 | F | ES | - |
| FJ670516 | F | ES | 2002 |
| DQ979025 | F | ES | - |
| FJ900267 | F | AO | 2006 |
| FJ900268 | F | AO | 2006 |
| FJ900266 | F | AO | 2006 |
| KJ849782 | F | BR | 2010 |
| EU446022 | F | DE | - |
| KJ849791 | F | BR | 2010 |
| GQ290462 | F | RU | 2008 |

**Subtype G**

| Accession number | Clade | Country | years |
| --- | --- | --- | --- |
| AY586547 | G | CU | 1999 |
| AY586548 | G | CU | 1999 |
| AY586549 | G | CU | 1999 |
| AB485662 | G | KE | 1993 |
| AF061641 | G | KE | 1993 |
| AY772535 | G | CM | 1996 |
| GU362882 | G | ES | 2009 |
| AF423760 | G | ES | 2000 |
| AF450098 | G | ES | 1999 |
| AY612637 | G | PT | - |
| FR846410 | G | PT | - |
| FJ670530 | G | ES | 2008 |
| FR846408 | G | PT | - |
| FJ670520 | G | ES | 2005 |
| FR846409 | G | PT | - |
| FJ389364 | G | CM | 2004 |
| AF061642 | G | SE | 1993 |
| FJ389366 | G | CM | 2004 |
| U88826 | G | NG | 1992 |
| AY371121 | G | CM | 2001 |
| AB231893 | G | GH | 2003 |
| FJ389367 | G | CM | 2001 |
| JN248584 | G | NG | 2009 |
| KJ948662 | G | ZA | 2001 |
| AF084936 | G | BE | 1996 |
| JN248593 | G | NG | 2009 |
| JN106043 | G | CN | 2008 |
| DQ168573 | G | NG | 2001 |
| DQ168579 | G | NG | 2001 |
| DQ168576 | G | NG | 2001 |
| DQ168575 | G | NG | 2001 |
| JN248586 | G | NG | 2009 |
| EU786670 | G | ES | 2005 |
| FJ389363 | G | CM | 2004 |
| JN248591 | G | NG | 2009 |
| JN248582 | G | NG | 2008 |
| FJ389365 | G | CM | 2004 |
| KF716477 | G | KE | 2009 |
| JX140676 | G | CM | 2010 |

**Subtype H**

| Accession number | Clade | Country | years |
| --- | --- | --- | --- |
| AF190127 | H | BE | 1993 |
| AF005496 | H | CF | 1990 |
| AF190128 | H | BE | 1993 |
| FJ711703 | H | GB | 2000 |

**Subtype J**

| Accession number | Clade | Country | years |
| --- | --- | --- | --- |
| AF082395 | J | SE | 1994 |
| AF082394 | J | SE | 1993 |
| GU237072 | J | CM | 2004 |

**Subtype K**

| AC number | Clade | Country | years |
| --- | --- | --- | --- |
| AJ249235 | K | CD | 1997 |
| AJ249239 | K | CM | 1996 |

**Table S2. Full genome genetic distance distributions observed within each corresponding clade.** Genetic distances are expressed in substitution/100 nucleotides.

| **Clade** | **Mean** | **Standard deviation** | **Median** | **Q25** | **Q75** |
| --- | --- | --- | --- | --- | --- |
| Group M | 15.3 | 2.3 | 16.0 | 15.1 | 16.7 |
| Group N | 6.9 | 0.8 | 7.0 | 6.5 | 7.4 |
| Group O | 12.0 | 2.9 | 11.4 | 10.0 | 14.5 |
| Group P | 4.8 | 2.0 | 5.6 | 5.5 | 6.0 |
| Subtype A | 9.2 | 1.7 | 9.5 | 8.0 | 10.8 |
| Subtype B/D | 12.7 | 1.1 | 12.7 | 12.1 | 13.4 |
| Subtype C | 8.4 | 0.6 | 8.4 | 7.9 | 9.1 |
| Subtype D | 9.4 | 1.9 | 9.3 | 8.0 | 10.9 |
| Subtype F | 9.3 | 2.0 | 9.3 | 8.3 | 10.5 |
| Subtype G | 8.8 | 1.4 | 8.9 | 7.9 | 9.7 |
| Subtype H | 8.6 | 0.9 | 8.8 | 8.5 | 9.2 |
| Subtype J | 5.9 | 2.7 | 7.3 | 5.0 | 7.4 |
| Subtype K | 8.3 | ND | 8.3 | ND | ND |
| Sub-subtype A1 | 8.3 | 1.0 | 8.4 | 7.7 | 9.1 |
| Sub-subtype A2 | 5.8 | 3.0 | 7.2 | 7.1 | 7.3 |
| Sub-subtype A3 | 8.2 | 2.6 | 9.3 | 8.5 | 9.7 |
| Sub-subtype A4 | 9.1 | 0.9 | 9.3 | 8.6 | 9.6 |
| Sub-subtype A6 | 3.5 | 1.2 | 3.3 | 2.7 | 4.2 |
| Sub-subtype A7 | 8.5 | ND | 8.5 | ND | ND |
| Subtype B | 8.4 | 1.3 | 8.4 | 8.0 | 9.6 |
| Sub-subtype D1 | 8.1 | 1.3 | 8.1 | 7.1 | 8.9 |
| Sub-subtype D2 | 8.1 | 1.8 | 8.3 | 7.0 | 9.2 |
| Sub-subtype D3 | 7.5 | 1.4 | 7.9 | 7.1 | 8.3 |
| Sub-subtype F1 | 8.5 | 1.4 | 8.6 | 7.8 | 9.4 |
| Sub-subtype F2 | 8.1 | 1.1 | 8.3 | 7.4 | 8.9 |

**Table S3. Net genetic divergence between each identified sub-subtypes.** The “net genetic divergence” between each identified sub-subtypes within corresponding subtypes, which also takes into account the within-sub-subtype diversity, was calculated as follow: if d_x,y_ is the average genetic divergence between two sub-subtypes, x and y, and d_x_ and d_y_ are the genetic diversities within populations x and y, respectively, net divergence, D_x,y_, is given by the expression D_x,y_ = d_x,y_ - (d_x_ + d_y_)/2.

**Previously described sub-subtypes**

| **d_A1_** | **d_A2_** | **d_A1,A2_** | **D_A1,A2_** |
| --- | --- | --- | --- |
| 9.6 | 7.3 | 12.7 | 4.25 |

| **d_B_** | **d_D_** | **d_B,D_** | **D_B,D_** |
| --- | --- | --- | --- |
| 8.8 | 9.5 | 12.7 | 3.55 |

| **d_F1_** | **d_F2_** | **d_F1,F2_** | **D_F1,F2_** |
| --- | --- | --- | --- |
| 8.9 | 8.8 | 12.7 | 3.85 |

**A sub-subtypes candidates**

| **d_A1_** | **d_A3_** | **d_A1,A3_** | **D_A1,A3_** |
| --- | --- | --- | --- |
| 8.3 | 8.2 | 9.9 | 1.65 |

| **d_A1_** | **d_A4_** | **d_A1,A4_** | **D_A1,A4_** |
| --- | --- | --- | --- |
| 8.3 | 9.1 | 11.7 | 3 |

| **d_A1_** | **d_A6_** | **d_A1,A6_** | **D_A1,A6_** |
| --- | --- | --- | --- |
| 8.3 | 3.5 | 10.8 | 4.9 |

| **d_A1_** | **d_A7_** | **d_A1,A7_** | **D_A1,A7_** |
| --- | --- | --- | --- |
| 8.3 | 8.5 | 11.4 | 3 |

| **d_A2_** | **d_A3_** | **d_A2,A3_** | **D_A2,A3_** |
| --- | --- | --- | --- |
| 5.8 | 8.2 | 12.2 | 5.2 |

| **d_A2_** | **d_A4_** | **d_A2,A4_** | **D_A2,A4_** |
| --- | --- | --- | --- |
| 5.8 | 9.1 | 12.5 | 5.05 |

| **d_A2_** | **d_A6_** | **d_A2,A6_** | **D_A2,A6_** |
| --- | --- | --- | --- |
| 5.8 | 3.5 | 12.7 | 8.05 |

| **d_A2_** | **d_A7_** | **d_A2,A7_** | **D_A2,A7_** |
| --- | --- | --- | --- |
| 5.8 | 8.5 | 13.3 | 6.15 |

| **d_A3_** | **d_A4_** | **d_A3,A4_** | **D_A3,A4_** |
| --- | --- | --- | --- |
| 8.2 | 9.1 | 11.5 | 2.85 |

| **d_A3_** | **d_A6_** | **d_A3,A6_** | **D_A3,A6_** |
| --- | --- | --- | --- |
| 8.2 | 3.5 | 10 | 4.15 |

| **d_A3_** | **d_A7_** | **d_A3,A7_** | **D_A3,A7_** |
| --- | --- | --- | --- |
| 8.2 | 8.5 | 10.9 | 2.55 |

**D sub-subtypes candidates**

| **d_A4_** | **d_A6_** | **d_A4,A6_** | **D_A4,A6_** |
| --- | --- | --- | --- |
| 9.1 | 3.5 | 11.8 | 5.5 |

| **d_A4_** | **d_A7_** | **d_A4,A7_** | **D_A4,A7_** |
| --- | --- | --- | --- |
| 9.1 | 8.5 | 12.5 | 3.7 |

| **d_A4_** | **d_A7_** | **d_A4,A7_** | **D_A4,A7_** |
| --- | --- | --- | --- |
| 9.1 | 8.5 | 12.5 | 3.7 |

| **d_D1_** | **d_D2_** | **d_D1,D2_** | **D_D1,D2_** |
| --- | --- | --- | --- |
| 8.1 | 8.1 | 10.8 | 2.7 |

| **d_D1_** | **d_D3_** | **d_D1,D3_** | **D_D1,D3_** |
| --- | --- | --- | --- |
| 8.1 | 7.5 | 10.9 | 3.1 |

| **d_D2_** | **d_D3_** | **d_D2,D3_** | **D_D2,D3_** |
| --- | --- | --- | --- |
| 8.1 | 7.5 | 11 | 3.2 |
